# Some Useful Integral Representations for Information-Theoretic Analyses

**DOI:** 10.3390/e22060707

**Published:** 2020-06-26

**Authors:** Neri Merhav, Igal Sason

**Affiliations:** The Andrew and Erna Viterbi Faculty of Electrical Engineering, Israel Institute of Technology Technion City, Haifa 3200003, Israel; merhav@ee.technion.ac.il

**Keywords:** integral representation, logarithmic expectation, moment-generating function, fractional moments, Rényi entropy, jamming, estimation errors, multivariate Cauchy distributions, guessing

## Abstract

This work is an extension of our earlier article, where a well-known integral representation of the logarithmic function was explored and was accompanied with demonstrations of its usefulness in obtaining compact, easily-calculable, exact formulas for quantities that involve expectations of the logarithm of a positive random variable. Here, in the same spirit, we derive an exact integral representation (in one or two dimensions) of the moment of a nonnegative random variable, or the sum of such independent random variables, where the moment order is a general positive non-integer real (also known as fractional moments). The proposed formula is applied to a variety of examples with an information-theoretic motivation, and it is shown how it facilitates their numerical evaluations. In particular, when applied to the calculation of a moment of the sum of a large number, *n*, of nonnegative random variables, it is clear that integration over one or two dimensions, as suggested by our proposed integral representation, is significantly easier than the alternative of integrating over *n* dimensions, as needed in the direct calculation of the desired moment.

## 1. Introduction

In mathematical analyses associated with many problems in information theory and related fields, one is often faced with the need to compute expectations of logarithmic functions of composite random variables (see, e.g., [1,2,3,4,5,6,7,8]), or moments of such random variables, whose order may be a general positive real, not even necessarily an integer (see, e.g., [9,10,11,12,13,14,15,16,17,18,19,20,21,22]).

In the case of the logarithmic function, the common practice is either to resort to approximate evaluations, provided by upper and lower bounds on the desired expression (for example, by using Jensen’s inequality) or to approximate the calculations by using the Taylor series expansion of the function lnx. More recently, it has become popular to use the replica trick (see, e.g., Chapter 8 in [23]), which is a non-rigorous, but useful technique, borrowed from statistical physics.

In our earlier work [6], we demonstrated how the following well-known integral representation of the logarithmic function,

(1) $$\begin{array}{c} \ln x=\int_{0}^{\infty }\left(e^{-u}-e^{-ux}\right)\;\frac{du}{u},\;x>0, \end{array}$$

can be useful in a variety of application areas in the field of information theory, including both source and channel coding, as well as other aspects of this field. To calculate the expectation, E{lnX}, where *X* is a positive random variable, the idea is simply to invoke the integral representation (1) and to commute the expectation and integration operators, i.e.,

(2) $$\begin{array}{c} E\left\{\ln X\right\}=\int_{0}^{\infty }(e^{-u}-E\left\{e^{-uX}\right\})\;\frac{du}{u}, \end{array}$$

thereby replacing the calculation of E{lnX} by the calculation of the moment-generating function (MGF), $M_{X}\left(u\right):=E\left\{e^{uX}\right\}$ for all u≤0, which is often much easier to express in closed form. Moreover, in frequently encountered situations where *X* is given by the sum of *n* independently identically distributed (i.i.d.) random variables, the MGF of *X* is given by the $n^{\mathrm{th}}$ power of the MGF of a single random variable in the sum that forms *X*. This reduces the dimension of the integration from *n* (in the original expression) to a single dimension of the integration over *u*. Interestingly, this integral representation has also been used in the statistical physics literature (see, e.g., [23] (p. 140) [24,25]), but not as much as the replica trick.

In this paper, we proceed in the same spirit as in [6], and we extend the scope to propose an integral representation of a general moment of a nonnegative random variable, *X*, namely the expectation, $E\{X^{\rho }\}$ for a given real ρ>0. Obviously, when ρ is an integer, this moment is simply given by the $\rho^{\mathrm{th}}$ order derivative of the MGF of *X*, calculated at the origin, as is very well known. However, the integral representation we propose, in this work, applies to any non-integer, positive ρ, and here too, it replaces the direct calculation of $E\{X^{\rho }\}$ by integration of an expression that involves the MGF of *X*. We refer to this representation as an extension of (2), as the latter can be obtained as a special case of the formula for $E\{X^{\rho }\}$, by invoking one of the equivalent identities

(3) $$\begin{array}{c} E\left\{\ln X\right\}=\lim_{\rho \to 0}\frac{E\{X^{\rho }\}-1}{\rho },\;E\left\{\ln X\right\}=\lim_{\rho \to 0}\frac{\ln[E\left\{X^{\rho }\right\}]}{\rho }. \end{array}$$

While the proposed integral representation of $E\{X^{\rho }\}$ can be readily obtained from [26] (p. 363, Identity (3.434.1)) in the range ρ∈(0,1), the nontrivial extension we propose for a non-integer and real ρ>1 is new to the best of our knowledge.

Fractional moments have been considered in the mathematical literature (see, e.g., [27,28,29,30]). A relationship between fractional and integer-order moments was considered in [27] by expressing a fractional moment as an infinite series, which depends on all the positive integer-order moments, followed by an algorithm for numerical calculations of fractional moments.

As in [6], the proposed integral representation is applied to a variety of examples with an information-theoretic motivation, and it is shown how it facilitates the numerical evaluations. In particular, similar to the case of the logarithmic function, when applied to the calculation of a moment of the sum of a large number, *n*, of nonnegative random variables, it is clear that integration over one or two dimensions, as suggested by our proposed integral representation, is significantly easier than the alternative of integrating over *n* dimensions, as needed in the direct calculation of the desired moment. Furthermore, single- or double-dimensional integrals can be instantly and accurately calculated using built-in numerical integration procedures.

Fractional moments have been considered in the mathematical literature (see, e.g., [27,28,29,30]). A relationship between fractional and integer-order moments was considered in [27] by expressing a fractional moment as an infinite series that depends on all the positive integer-order moments, which was followed by an algorithm for numerical calculations of fractional moments.

The outline of the remainder part of this paper is as follows. In Section 2, we provide the mathematical background associated with the integral representation in general. In Section 3, we demonstrate this integral representation in applications, including: moments of guesswork, moments of estimation errors, differential Rényi entropies of generalized multivariate Cauchy distributions, and mutual information calculations of a certain model of a jammed channel. Each one of these examples occupies one subsection of Section 3. The integral representations in this paper are not limited to the examples in Section 3, and such representations can be proven useful in other information-theoretic problems (see, e.g., [6] and the references therein for the integral representation of the logarithmic expectation and some of its information-theoretic applications).

## 2. Statistical Moments of Arbitrary Positive Orders

It is well known that integer-order moments of a random variable *X* are calculable from its MGF

(4) $$\begin{array}{c} M_{X}\left(u\right):=E\left\{e^{uX}\right\},\;u\in R, \end{array}$$

by using its derivatives, calculated at u=0, i.e.,

(5) $$\begin{array}{c} E\left\{X^{\rho }\right\}=M_{X}^{\left(\rho \right)}\left(0\right),\;\rho \in N. \end{array}$$

Quite often, however, there is a theoretical and practical interest to calculate fractional moments of nonnegative random variables. We next obtain a closed-form integral expression of the $\rho^{\mathrm{th}}$ moment of a nonnegative random variable *X*, as a functional of its MGF, for any positive real ρ. Before we proceed, it should be noted that for ρ∈(0,1), such an expression is available in handbooks of standard tables of integrals, for example in [26] (p. 363, Identity (3.434.1)). The first innovation here, however, is in a nontrivial extension of this formula for all ρ>0 as an expression that involves a one-dimensional integral. It should be noted that although the definition of a fractional moment of a random variable (RV) is also given by a one-dimensional integral (or a sum, depending on whether the RV is discrete or continuous), the utility of our formula is, e.g., in expressing the $\rho^{\mathrm{th}}$ moment of a sum of nonnegative and independent random variables as a one-dimensional integral, instead of an *n*-dimensional integral, which is obtained by the direct definition. This new formula serves as the basic building block in all of our information-theoretic applications throughout this paper.

We first define the Beta and Gamma functions (see, e.g., Section 8.3 in [26] and Chapter 5 in [31]):

(6) $$\begin{array}{cc} B(u,v) & :=\int_{0}^{1}t^{u-1}{\left(1-t\right)}^{v-1}\;dt,\;u,v>0, \end{array}$$

(7) $$\begin{array}{cc} \Gamma (u) & :=\int_{0}^{\infty }t^{u-1}e^{-t}\;dt,\;u>0, \end{array}$$

where these functions are related by the equality

(8) $$\begin{array}{c} B\left(u,v\right)=\frac{\Gamma (u)\;\Gamma (v)}{\Gamma (u+v)},\;u,v>0. \end{array}$$

**Theorem** **1.** *Let X be a nonnegative random variable, and let ρ>0 be a non-integer real. Then,*

(9) $$\begin{array}{cc} E\{X^{\rho }\} & =\frac{1}{1+\rho }\;\sum_{\ell =0}^{\left\lfloor \rho \right\rfloor }\frac{\alpha_{\ell }}{B(\ell +1,\rho +1-\ell )}\\ \; & \;+\frac{\rho \;\sin(\pi \rho )\;\Gamma (\rho )}{\pi }\int_{0}^{\infty }\frac{1}{u^{\rho +1}}\;\left(\;\sum_{j=0}^{\left\lfloor \rho \right\rfloor }\left\{\frac{{\left(-1\right)}^{j}\;\alpha_{j}}{j!}\;u^{j}\right\}\;e^{-u}-M_{X}\left(-u\right)\right)\;du, \end{array}$$

*where, for all j∈{0,1,…,},*

(10) $$\begin{array}{c} \alpha_{j}:=E\{{\left(X-1\right)}^{j}\} \end{array}$$

(11) $$\begin{array}{cc} \; & =\frac{1}{j+1}\sum_{\ell =0}^{j}\frac{{\left(-1\right)}^{j-\ell }\;M_{X}^{\left(\ell \right)}\left(0\right)}{B(\ell +1,j-\ell +1)}. \end{array}$$

**Proof.**  See Appendix A. □

**Remark** **1.** *The proof of (9) in Appendix A does not apply to ρ∈N (see (A7) and (A8) etc., where the denominators vanish for ρ∈N). In the latter case, by referring to the second term on the right-hand side of (9), we get sin(πρ)=0, and also, the integral diverges (specifically, for ρ∈N, the integrand scales like $\frac{1}{u}$ for u that is sufficiently close to zero), yielding an expression of the type 0·∞. However, taking a limit in (9) where we let ρ tend to an integer and applying L’Hôpital’s rule can reproduce the well-known result in (5).*

**Corollary** **1.** *For any ρ∈(0,1),*

(12) $$\begin{array}{cc} E\{X^{\rho }\} & =1+\frac{\rho }{\Gamma (1-\rho )}\int_{0}^{\infty }\frac{e^{-u}-M_{X}\left(-u\right)}{u^{1+\rho }}\;du. \end{array}$$

**Proof.**  Equation (12) is due to Theorem 1 and by using (A20) and (A22) (see Appendix A), and $\alpha_{0}:=1$, which give

(13) $$\begin{array}{cc} \Gamma (\rho )\;\Gamma (1-\rho ) & =\frac{\pi }{\sin(\pi \rho )}, \end{array}$$

(14) $$\begin{array}{cc} \frac{1}{1+\rho }\;\frac{\alpha_{0}}{B(1,\rho +1)} & =\frac{1}{1+\rho }\;\frac{\Gamma (\rho +2)}{\Gamma (\rho +1)}\\ \; & =1. \end{array}$$

 □

**Remark** **2.** *Corollary 1 also follows from [26] (p. 363, Identity (3.434.1)) (see Section 4 in [6]).*

**Corollary** **2.** *[6] Let X be a positive random variable. Then,*

(15) $$\begin{array}{c} E\left\{\ln X\right\}=\int_{0}^{\infty }\frac{e^{-u}-M_{X}\left(-u\right)}{u}\;du. \end{array}$$

A proof of (15) was presented in Section 2 in [6], based on the integral representation of the logarithmic function in (1), and by interchanging the integration and the expectation (due to Fubini’s theorem). It can be alternatively proven by using Corollary 1, the identity

(16) $$\begin{array}{c} \ln x=\lim_{\rho \to 0}\frac{x^{\rho }-1}{\rho },\;x>0, \end{array}$$

and swapping the order of the expectation and limit by the dominated convergence theorem.

Identity (15) has many useful information-theoretic applications in its own right, as demonstrated in [6], and here, we add even some more. The current work is an extension and further development of [6], whose main theme is exploiting Theorem 1 and studying its information-theoretic applications, as well as some more applications of the logarithmic expectation.

## 3. Applications

In this section, we exemplify the usefulness of the integral representation of the $\rho^{\mathrm{th}}$ moment in Theorem 1 and the logarithmic expectation in several problem areas in information theory and statistics. These include analyses of randomized guessing, estimation errors, Rényi entropy of *n*-dimensional generalized Cauchy distributions, and finally, calculations of the mutual information for channels with a certain jammer model. To demonstrate the direct computability of the relevant quantities, we also present graphs of their numerical calculations.

### 3.1. Moments of Guesswork

Consider the problem of guessing the realization of a random variable, which takes on values in a finite alphabet, using a sequence of yes/no questions of the form “Is $X=x_{1}$?”, “Is $X=x_{2}$?”, etc., until a positive response is provided by a party that observes the actual realization of *X*. Given a distribution of *X*, a commonly used performance metric for this problem is the expected number of guesses or, more generally, the $\rho^{\mathrm{th}}$ moment of the number of guesses until *X* is guessed successfully. When it comes to guessing random vectors, say, of length *n*, minimizing the moments of the number of guesses by different (deterministic or randomized) guessing strategies has several applications and motivations in information theory, such as sequential decoding, guessing passwords, etc., and it is also strongly related to lossless source coding (see, e.g., [9,10,11,12,13,19,20,21,22,32,33,34]). In this vector case, the moments of the number of guesses behave as exponential functions of the vector dimension, *n*, at least asymptotically, as *n* grows without bound. For random vectors with i.i.d. components, the best achievable asymptotic exponent of the $\rho^{\mathrm{th}}$ guessing moment is expressed in [9] by using the Rényi entropy of *X* of order $\tilde{\rho }:=\frac{1}{1+\rho }$. Arikan assumed in [9] that the distribution of *X* is known and analyzed the optimal deterministic guessing strategy, which orders the guesses according to nonincreasing probabilities. Refinements of the exponential bounds in [9] with tight upper and lower bounds on the guessing moments for optimal deterministic guessing were recently derived in [19]. In the sequel, we refer to randomized guessing strategies, rather than deterministic strategies, and we aim to derive exact, calculable expressions for their associated guessing moments (as is later explained in this subsection).

Let the random variable *X* take on values in a finite alphabet X. Consider a random guessing strategy where the guesser sequentially submits a sequence of independently drawn random guesses according to a certain probability distribution, $\tilde{P}\left(\cdot \right)$, defined on X. Randomized guessing strategies have the advantage that they can be used by multiple asynchronous agents, which submit their guesses concurrently (see [33,34]).

In this subsection, we consider the setting of randomized guessing and obtain an exact representation of the guessing moment in the form of a one-dimensional integral. Let x∈X be any realization of *X*, and let the guessing distribution, $\tilde{P}$, be given. The random number, *G*, of independent guesses until success has the geometric distribution

(17) $$\begin{array}{c} \mathrm{Pr}\left\{G=k|x\right\}=[1-\tilde{P}\left(x\right){]}^{k-1}\;\tilde{P}\left(x\right),\;k\in N, \end{array}$$

and so, the corresponding MGF is equal to

(18) $$\begin{array}{cc} M_{G}\left(u|x\right) & =\sum_{k=1}^{\infty }e^{ku}\;\mathrm{Pr}\left\{G=k|x\right\}\\ \; & =\frac{\tilde{P}\left(x\right)}{e^{-u}-(1-\tilde{P}\left(x\right))},\;u<\ln\frac{1}{1-\tilde{P}\left(x\right)}. \end{array}$$

In view of (9)–(11) and (18), for x∈X and non-integer ρ>0,

(19) $$\begin{array}{cc} E\{G^{\rho }|x\} & =\frac{1}{1+\rho }\;\sum_{\ell =0}^{\left\lfloor \rho \right\rfloor }\frac{\alpha_{\ell }}{B(\ell +1,\rho +1-\ell )}\\ \; & \;+\frac{\rho \;\sin(\pi \rho )\;\Gamma (\rho )}{\pi }\int_{0}^{\infty }\frac{1}{u^{\rho +1}}\;\left(\;\sum_{j=0}^{\left\lfloor \rho \right\rfloor }\left\{\frac{{\left(-1\right)}^{j}\;\alpha_{j}}{j!}\;u^{j}\right\}\;e^{-u}-\frac{\tilde{P}\left(x\right)}{e^{u}-(1-\tilde{P}\left(x\right))}\right)\;du, \end{array}$$

with $\alpha_{0}:=1$, and for all j∈N

(20) $$\begin{array}{cc} \alpha_{j} & :=E\left\{{\left(G-1\right)}^{j}|X=x\right\}\\ \; & =\sum_{k=1}^{\infty }{\left(k-1\right)}^{j}\;{\left(1-\tilde{P}\left(x\right)\right)}^{k-1}\;\tilde{P}\left(x\right)\\ \; & =\tilde{P}\left(x\right)\;{\mathrm{Li}}_{-j}\left(1-\tilde{P}\left(x\right)\right). \end{array}$$

In (20), ${\mathrm{Li}}_{-j}\left(\cdot \right)$ is a polylogarithm (see, e.g., Section 25.12 in [31]), which is given by

(21) $$\begin{array}{c} {\mathrm{Li}}_{-j}\left(x\right)={\left(x\;\frac{d}{dx}\right)}^{j}\;\frac{x}{1-x},\;\forall \;j\in N\cup \left\{0\right\}, \end{array}$$

with ${\left(x\;\frac{d}{dx}\right)}^{j}$ denoting differentiation with respect to *x* and multiplication of the derivative by *x*, repeatedly *j* times. In particular, we have

(22) $$\begin{array}{c} {\mathrm{Li}}_{0}\left(x\right)=\frac{x}{1-x},\;{\mathrm{Li}}_{-1}\left(x\right)=\frac{x}{{\left(1-x\right)}^{2}},\;{\mathrm{Li}}_{-2}\left(x\right)=\frac{x(1+x)}{{\left(1-x\right)}^{3}}, \end{array}$$

and so on. The function ${\mathrm{Li}}_{-j}\left(x\right)$ is a built-in function in the MATLAB and Mathematica software, which is expressed as polylog(−j,x). By Corollary 1, if ρ∈(0,1), then (19) is simplified to

(23) $$\begin{array}{cc} E\{G^{\rho }|x\} & =1+\frac{\rho }{\Gamma (1-\rho )}\int_{0}^{\infty }\frac{e^{-u}-e^{-2u}}{u^{\rho +1}[{\left(1-\tilde{P}\left(x\right)\right)}^{-1}-e^{-u}]}\;du. \end{array}$$

Let *P* denote the distribution of *X*. Averaging over *X* to get the unconditional $\rho^{\mathrm{th}}$ moment using (23), one obtains for all ρ∈(0,1),

(24) $$\begin{array}{c} E\left\{G^{\rho }\right\}=1+\frac{\rho }{\Gamma (1-\rho )}\int_{0}^{1}\frac{1-z}{{\left(-\ln z\right)}^{\rho +1}}\sum_{x\in X}\frac{P\left(x\right)(1-\tilde{P}\left(x\right))}{1-z(1-\tilde{P}\left(x\right))}\;dz, \end{array}$$

where (24) is obtained by using the substitution $z:=e^{-u}$. A suitable expression of such an integral is similarly obtained, for all ρ>0, by averaging (19) over *X*. In comparison, a direct calculation of the $\rho^{\mathrm{th}}$ moment gives

(25) $$\begin{array}{c} E\left\{G^{\rho }\right\}=\sum_{x\in X}P\left(x\right)\;E\left\{G^{\rho }|x\right\}=\sum_{k=1}^{\infty }\sum_{x\in X}k^{\rho }{\left(1-\tilde{P}\left(x\right)\right)}^{k-1}\;\tilde{P}\left(x\right)\;P\left(x\right). \end{array}$$

The double sum in (25) involves a numerical computation of an infinite series, where the number of terms required to obtain a good approximation increases with ρ and needs to be determined. The right-hand side of (24), on the other hand, involves integration over [0,1]. For every practical purpose, however, definite integrals in one or two dimensions can be calculated instantly using built-in numerical integration procedures in MATLAB, Maple, Mathematica, or any other mathematical software tools, and the computational complexity of the integral in (24) is not affected by ρ.

As a complement to (19) (which applies to a non-integral and positive ρ), we obtain that the $\rho^{\mathrm{th}}$ moment of the number of randomized guesses, with ρ∈N, is equal to

(26) E{Gρ|x}=∑j=0ρρjE{G−1j|x}=∑j=0ρρjαj=1+P˜(x)∑j=1ρρjLi−j(1−P˜(x)),

where (26) follows from (20) and since $\alpha_{0}=1$. By averaging over *X*,

(27) E{Gρ}=1+∑x∈cXP(x)P˜(x)∑j=1ρρjLi−j(1−P˜(x)).

To conclude, (19) and its simplification in (23) for ρ∈(0,1) give calculable one-dimensional integral expressions for the $\rho^{\mathrm{th}}$ guessing moment with any ρ>0. This refers to a randomized guessing strategy whose practical advantages were further explained in [33,34]. This avoids the need for numerical calculations of infinite sums. A further simplification for ρ∈N is provided in (26) and (27), expressed in closed form as a function of polylogarithms.

### 3.2. Moments of Estimation Errors

Let $X_{1},\ldots ,X_{n}$ be i.i.d. random variables with an unknown expectation θ to be estimated, and consider the simple estimator,

(28) $$\begin{array}{c} {\hat{\theta }}_{n}=\frac{1}{n}\sum_{i=1}^{n}X_{i}. \end{array}$$

For given ρ>0, we next derive an easily-calculable expression of the $\rho^{\mathrm{th}}$ moment of the estimation error.

Let $D_{n}:={\left({\hat{\theta }}_{n}-\theta \right)}^{2}$ and $\rho^{\prime }:=\frac{\rho }{2}$. By Theorem 1, if ρ>0 is a non-integral multiple of two, then

(29) $$\begin{array}{cc} E\left\{{\left|{\hat{\theta }}_{n}-\theta \right|}^{\rho }\right\}\; & =E\left\{D_{n}^{\rho^{\prime }}\right\}\\ \; & =\frac{2}{2+\rho }\;\sum_{\ell =0}^{\left\lfloor \rho /2\right\rfloor }\frac{\alpha_{\ell }}{B\left(\ell +1,\rho /2+1-\ell \right)} \end{array}$$

(30) $$\begin{array}{cc} & \;+\frac{\rho }{2\pi }\;\sin\left(\frac{\pi \rho }{2}\right)\;\Gamma \left(\frac{\rho }{2}\right)\int_{0}^{\infty }\frac{1}{u^{\rho /2+1}}\;\left(\;\sum_{j=0}^{\left\lfloor \rho /2\right\rfloor }\left\{\frac{{\left(-1\right)}^{j}\;\alpha_{j}}{j!}\;u^{j}\right\}\;e^{-u}-M_{D_{n}}\left(-u\right)\right)\;du, \end{array}$$

where

(31) $$\begin{array}{c} M_{D_{n}}\left(-u\right)=E\{\exp(-u{\left({\hat{\theta }}_{n}-\theta \right)}^{2})\},\;\forall \;u\geq 0, \end{array}$$

$\alpha_{0}:=1$, and for all j∈N (see (11))

(32) $$\begin{array}{c} \alpha_{j}=\frac{1}{j+1}\sum_{\ell =0}^{j}\frac{{\left(-1\right)}^{j-\ell }\;M_{D_{n}}^{\left(\ell \right)}\left(0\right)}{B(\ell +1,j-\ell +1)}. \end{array}$$

By Corollary 1 and (29), if in particular ρ∈(0,2), then the right-hand side of (30) is simplified to

(33) $$\begin{array}{c} E\{{\left|{\hat{\theta }}_{n}-\theta \right|}^{\rho }\}=1+\frac{\rho }{2\;\Gamma (1-\frac{1}{2}\;\rho )}\int_{0}^{\infty }u^{-(1+\frac{1}{2}\rho )}\;\left[e^{-u}-M_{D_{n}}\left(-u\right)\right]\;du, \end{array}$$

and, for all k∈N,

(34) $$\begin{array}{cc} \; & E\{|{\hat{\theta }}_{n}{-\theta |}^{2k}\}=M_{D_{n}}^{\left(k\right)}\left(0\right). \end{array}$$

In view of (29)–(34), obtaining a closed-form expression for the $\rho^{\mathrm{th}}$ moment of the estimation error, for an arbitrary ρ>0, hinges on the calculation of the right side of (31) for all u≥0. To this end, we invoke the identity

(35) $$\begin{array}{c} e^{-uz^{2}}=\frac{1}{2\sqrt{\pi u}}\int_{-\infty }^{\infty }e^{-j\omega z-\omega^{2}/\left(4u\right)}\;d\omega ,\;\forall \;u>0,\;z\in R, \end{array}$$

which is the MGF of a zero-mean Gaussian random variable with variance $\frac{1}{2u}$. Together with (31), it gives (see Appendix B.1)

(36) $$\begin{array}{c} M_{D_{n}}\left(-u\right)=\frac{1}{2\sqrt{\pi u}}\int_{-\infty }^{\infty }e^{-j\omega \theta }\;\phi_{X}^{n}\left(\frac{\omega }{n}\right)\;e^{-\omega^{2}/\left(4u\right)}\;d\omega ,\;\forall \;u>0, \end{array}$$

where *X* is a generic random variable with the same distribution as $X_{i}$ for all *i*.

The combination of (30)–(34) enables calculating exactly the $\rho^{\mathrm{th}}$ moment $E\{|{\hat{\theta }}_{n}{-\theta |}^{\rho }\}$, for any given ρ>0, in terms of a two-dimensional integral. Combining (33) and (36) yields, for all ρ∈(0,2),

(37) $$\begin{array}{cc} \; & E\{{\left|{\hat{\theta }}_{n}-\theta \right|}^{\rho }\}\\ \; & =1+\frac{\rho }{2\;\Gamma (1-\frac{1}{2}\;\rho )}\int_{0}^{\infty }\int_{-\infty }^{\infty }u^{-(\rho /2+1)}\left[\frac{1}{2}\;e^{-u-|\omega |}-\frac{1}{2\sqrt{\pi u}}\;\phi_{X}^{\;n}\left(\frac{\omega }{n}\right)\;e^{-j\omega \theta -\omega^{2}/\left(4u\right)}\right]\;d\omega \;du, \end{array}$$

where we have used the identity $\int_{-\infty }^{\infty }\frac{1}{2}\;e^{-|\omega |}\;d\omega =1$ in the derivation of the first term of the integral on the right-hand side of (37).

As an example, consider the case where ${\left\{X_{i}\right\}}_{i=1}^{n}$ are i.i.d. Bernoulli random variables with

(38) $$\begin{array}{c} P\left\{X_{1}=1\right\}=\theta ,\;P\left\{X_{1}=0\right\}=1-\theta \end{array}$$

where the characteristic function is given by

(39) $$\begin{array}{c} \phi_{X}\left(u\right):=E\left\{e^{juX}\right\}=1+\theta \left(e^{ju}-1\right),\;u\in R. \end{array}$$

Thanks to the availability of the exact expression, we can next compare the exact $\rho^{\mathrm{th}}$ moment of the estimation error $|{\hat{\theta }}_{n}-\theta |$, with the following closed-form upper bound (see Appendix B.2) and thereby assess its tightness:

(40) $$\begin{array}{c} E\{{\left|{\hat{\theta }}_{n}-\theta \right|}^{\rho }\}\leq K\left(\rho ,\theta \right)\cdot n^{-\rho /2}, \end{array}$$

which holds for all n∈N, ρ>0, and θ∈[0,1], with

(41) $$\begin{array}{c} K\left(\rho ,\theta \right):=\rho \;\Gamma \left(\frac{\rho }{2}\right)\;{\left(2\theta \;(1-\theta )\right)}^{\rho /2}. \end{array}$$

**Figure 1:**
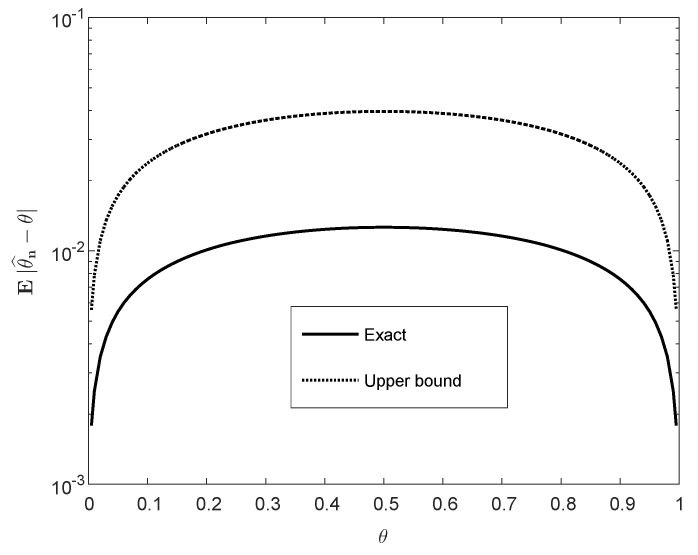
$E\;\left|{\hat{\theta }}_{n}-\theta \right|$ (see (37) and (39)) versus its upper bound in (40) as functions of θ∈[0,1] with n=1000.

**Figure 2:**
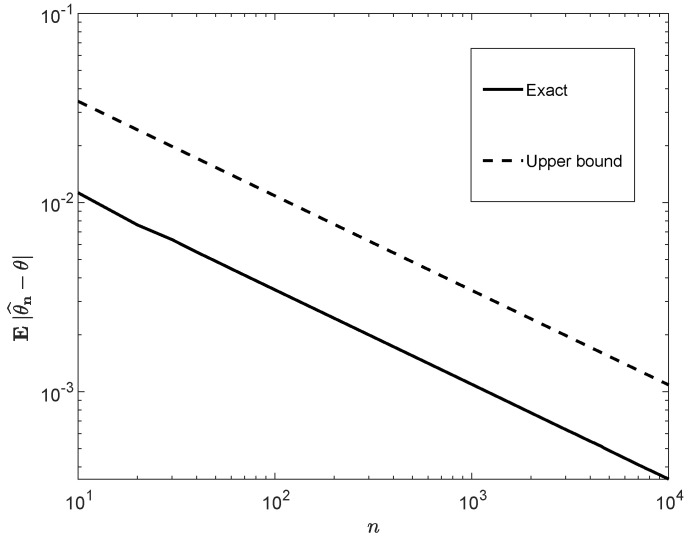
$E\left|{\hat{\theta }}_{n}-\theta \right|$ (see (37) and (39)) versus its upper bound in (40) as functions of *n* with $\theta =\frac{1}{4}$.

Figure 1 and Figure 2 display plots of $E\;|{\hat{\theta }}_{n}-\theta |$ as a function of θ and *n*, in comparison to the upper bound (40). The difference in the plot of Figure 1 is significant except for the boundaries of the interval [0,1], where both the exact value and the bound vanish. Figure 2 indicates that the exact value of $E\;|{\hat{\theta }}_{n}-\theta |$, for large *n*, scales like $\sqrt{n}$; this is reflected by the apparent parallelism of the curves in both graphs and by the upper bound (40).

To conclude, this subsection provides an exact, double-integral expression for the $\rho^{\mathrm{th}}$ moment of the estimation error of the expectation of *n* i.i.d. random variables. In other words, the dimension of the integral does not increase with *n*, and it is a calculable expression. We further compare our expression with an upper bound that stems from concentration inequalities. Although the scaling of the bound as a polynomial of *n* is correct, the difference between the exact expression and the bound is significant (see Figure 1 and Figure 2).

### 3.3. Rényi Entropy of Extended Multivariate Cauchy Distributions

Generalized Cauchy distributions, their mathematical properties, and applications are of interest (see, e.g., [6,35,36,37]). The Shannon differential entropy of a family of generalized Cauchy distributions was derived in Proposition 1 in [36], and also, a lower bound on the differential entropy of a family of extended multivariate Cauchy distributions (cf. Equation (42) in [37]) was derived in Theorem 6 in [37]. Furthermore, an exact single-letter expression for the differential entropy of the different family of extended multivariate Cauchy distributions was recently derived in Section 3.1 in [6]. Motivated by these studies, as well as the various information-theoretic applications of Rényi information measures, we apply Theorem 1 to obtain the Rényi (differential) entropy of an arbitrary positive order α for the extended multivariate Cauchy distributions in Section 3.1 in [6]. As we shall see in this subsection, the integral representation for the Rényi entropy of the latter family of extended multivariate Cauchy distributions is two-dimensional, irrespective of the dimension *n* of the random vector.

Let $X^{n}=\left(X_{1},\ldots ,X_{n}\right)$ be a random vector whose probability density function is of the form

(42) $$\begin{array}{c} f\left(x^{n}\right)=\frac{C_{n}}{{\left[1+\sum_{i=1}^{n}g\left(x_{i}\right)\right]}^{q}},\;x^{n}=\left(x_{1},\ldots ,x_{n}\right)\in R^{n}, \end{array}$$

for a certain function g:R→[0,∞) and a positive constant *q* such that

(43) $$\begin{array}{c} \int_{R^{n}}\frac{1}{{\left[1+\sum_{i=1}^{n}g\left(x_{i}\right)\right]}^{q}}\;dx^{n}<\infty . \end{array}$$

We refer to this kind of density (see also Section 3.1 in [6]) as a generalized multivariate Cauchy density because the multivariate Cauchy density function is the special case pertaining to the choices $g\left(x\right)=x^{2}$ and $q=\frac{1}{2}\left(n+1\right)$. The differential Shannon entropy of the generalized multivariate Cauchy density was derived in Section 3.1 in [6] using the integral representation of the logarithm (1), where it was presented as a two-dimensional integral.

We next extend the analysis of [6] to differential Rényi entropies of an arbitrary positive order α (recall that the differential Rényi entropy is specialized to the differential Shannon entropy at α=1 [38]). We show that, for the generalized multivariate Cauchy density, the differential Rényi entropy can be presented as a two-dimensional integral, rather than an *n*-dimensional integral. Defining

(44) $$\begin{array}{c} Z\left(t\right):=\int_{-\infty }^{\infty }e^{-tg(x)}\;dx,\;t>0, \end{array}$$

we get from (42) (see Section 3.1 in [6]) that

(45) $$\begin{array}{c} C_{n}=\frac{\Gamma (q)}{\int_{0}^{\infty }t^{q-1}e^{-t}Z^{n}\left(t\right)\;dt}. \end{array}$$

For $g\left(x\right)={\left|x\right|}^{\theta }$, with a fixed θ>0, (44) implies that

(46) $$\begin{array}{c} Z\left(t\right)=\frac{2\;\Gamma (1/\theta )}{\theta \;t^{1/\theta }}. \end{array}$$

In particular, for θ=2 and $q=\frac{1}{2}\left(n+1\right)$, we get the multivariate Cauchy density from (42). In this case, it follows from (46) that $Z\left(t\right)=\sqrt{\frac{\pi }{t}}$ for t>0, and from (45)

(47) $$\begin{array}{c} C_{n}=\frac{\Gamma \left(\frac{n+1}{2}\right)}{\pi^{(n+1)/2}}. \end{array}$$

For α∈(0,1)∪(1,∞), the (differential) Rényi entropy of order α is given by

(48) $$\begin{array}{cc} h_{\alpha }\left(X^{n}\right) & :=\frac{1}{1-\alpha }\;\log\int_{R^{n}}f^{\alpha }\left(x^{n}\right)\;dx^{n}\\ \; & \;=\frac{1}{1-\alpha }\;\log E\left[f^{\alpha -1}\left(X^{n}\right)\right]. \end{array}$$

Using the Laplace transform relation,

(49) 1sq=1Γ(q)∫0∞tq−1e−stdt,∀q>0,Re(s)>0,

we obtain that, for α>1 (see Appendix C),

(50) $$\begin{array}{cc} h_{\alpha }\left(X^{n}\right) & =\frac{\alpha }{\alpha -1}\;\log\int_{0}^{\infty }t^{q-1}e^{-t}Z^{n}\left(t\right)\;dt+\frac{\log\Gamma \left(q(\alpha -1)\right)}{\alpha -1}-\log\Gamma \left(q\right)\\ \; & \;-\frac{1}{\alpha -1}\;\log\int_{0}^{\infty }\int_{0}^{\infty }t^{q(\alpha -1)-1}u^{q-1}e^{-(t+u)}\;Z^{n}\left(t+u\right)\;du\;dt. \end{array}$$

If α∈(0,1), we distinguish between the following two cases:

(a) If $\alpha =1-\frac{m}{q}$ for some m∈{1,…,q−1}, then
  (51) $$\begin{array}{c} h_{\alpha }\left(X^{n}\right)=\frac{\alpha \;\log C_{n}}{1-\alpha }-\frac{\log\Gamma (q)}{1-\alpha }+\frac{1}{1-\alpha }\;\log\left(\sum_{\ell =0}^{m}\left\{{\left(-1\right)}^{m-\ell }\int_{0}^{\infty }t^{q-1}e^{-t}\varphi_{n}^{\left(\ell \right)}\left(t\right)\;dt\right\}\right), \end{array}$$
  with
  (52) $$\begin{array}{c} \varphi_{n}\left(t\right):=Z^{n}\left(t\right),\;\forall \;t\geq 0. \end{array}$$
(b) Otherwise (i.e., if ρ:=q(1−α)∉N), then
  (53) $$\begin{array}{cc} h_{\alpha }\left(X^{n}\right) & =-\log C_{n}+\frac{1}{1-\alpha }\;\log(\frac{1}{1+\rho }\;\sum_{\ell =0}^{\left\lfloor \rho \right\rfloor }\frac{\beta_{\ell }\left(n\right)}{B(\ell +1,\rho +1-\ell )}+\frac{\rho \;\sin(\pi \rho )\;\Gamma (\rho )}{\pi }\\ \; & \;\cdot \int_{0}^{\infty }\frac{e^{-u}}{u^{\rho +1}}\;\left(\;\sum_{j=0}^{\left\lfloor \rho \right\rfloor }\left\{\frac{{\left(-1\right)}^{j}\;\beta_{j}\left(n\right)}{j!}\;u^{j}\right\}-\frac{C_{n}}{\Gamma (q)}\int_{0}^{\infty }t^{q-1}e^{-t}Z^{n}\left(t+u\right)\;dt\right)\;du), \end{array}$$
  where $\beta_{0}:=1$, and for all j∈N,
  (54) βj(n):=CnΓ(q)∑ℓ=0j(−1)j−ℓB(ℓ+1,j−ℓ+1)∑k=0ℓ(−1)ℓ−kℓk∫0∞tq−1e−tφn(k)(t)dt.

The proof of the integral expressions of the Rényi entropy of order α∈(0,1), as given in (50)–(54), is provided in Appendix C.

Once again, the advantage of these expressions, which do not seem to be very simple (at least on the face of it), is that they only involve one- or two-dimensional integrals, rather than an expression of an *n*-dimensional integral (as it could have been in the case of an *n*-dimensional density).

### 3.4. Mutual Information Calculations for Communication Channels with Jamming

Consider a channel that is fed by an input vector $X^{n}=\left(X_{1},\ldots ,X_{n}\right)\in X^{n}$ and generates an output vector $Y^{n}=\left(Y_{1},\ldots ,Y_{n}\right)\in Y^{n}$, where X and Y are either finite, countably infinite, or continuous alphabets, and $X^{n}$ and $Y^{n}$ are their $n^{\mathrm{th}}$ order Cartesian powers. Let the conditional probability distribution of the channel be given by

(55) $$\begin{array}{c} p_{Y^{n}|X^{n}}\left(y^{n}|x^{n}\right)=\frac{1}{n}\sum_{i=1}^{n}\left\{\prod_{j\neq i}q_{Y|X}\left(y_{j}|x_{j}\right)\;r_{Y|X}\left(y_{i}|x_{i}\right)\right\}, \end{array}$$

where $r_{Y|X}\left(\cdot |\cdot \right)$ and $q_{Y|X}\left(\cdot |\cdot \right)$ are given conditional probability distributions of *Y* given *X*, $x^{n}=\left(x_{1},\ldots ,x_{n}\right)\in X^{n}$ and $y^{n}=\left(y_{1},\ldots ,y_{n}\right)\in Y^{n}$. This channel model refers to a discrete memoryless channel (DMC), which is nominally given by

(56) $$\begin{array}{c} q_{Y^{n}|X^{n}}\left(y^{n}|x^{n}\right)=\prod_{i=1}^{n}q_{Y|X}\left(y_{i}|x_{i}\right), \end{array}$$

where one of the transmitted symbols is jammed at a uniformly distributed random time, *i*, and the transition distribution of the jammed symbol is given by $r_{Y|X}\left(y_{i}|x_{i}\right)$ instead of $q_{Y|X}\left(y_{i}|x_{i}\right)$. The restriction to a single jammed symbol is made merely for the sake of simplicity, but it can easily be extended.

We wish to evaluate how the jamming affects the mutual information $I(X^{n};Y^{n})$. Clearly, when one talks about jamming, the mutual information is decreased, but this is not part of the mathematical model, where the relation between *r* and *q* has not been specified. Let the input distribution be given by the product form

(57) $$\begin{array}{c} p_{X^{n}}\left(x^{n}\right)=\prod_{i=1}^{n}p_{X}\left(x_{i}\right),\;x^{n}\in X^{n}. \end{array}$$

The mutual information (in nats) is given by

(58) $$\begin{array}{ll} I(X^{n};Y^{n}) & =h\left(Y^{n}\right)-h\left(Y^{n}|X^{n}\right) \end{array}$$

(59) $$\begin{array}{ll} \; & =\int_{X^{n}\times Y^{n}}p_{X^{n},Y^{n}}\left(x^{n},y^{n}\right)\;\ln p_{Y^{n}|X^{n}}\left(y^{n}|x^{n}\right)\;dx^{n}\;dy^{n}-\int_{Y^{n}}p_{Y^{n}}\left(y^{n}\right)\;\ln p_{Y^{n}}\left(y^{n}\right)\;dy^{n}. \end{array}$$

For the simplicity of notation, we henceforth omit the domains of integration whenever they are clear from the context. We have,

(60) $$\begin{array}{cc} \; & \;\int p_{X^{n},Y^{n}}\left(x^{n},y^{n}\right)\;\ln p_{Y^{n}|X^{n}}\left(y^{n}|x^{n}\right)\;dx^{n}\;dy^{n}\\ \; & =\int p_{X^{n},Y^{n}}\left(x^{n},y^{n}\right)\;\ln\left(\frac{p_{Y^{n}|X^{n}}\left(y^{n}|x^{n}\right)}{q_{Y^{n}|X^{n}}\left(y^{n}|x^{n}\right)}\right)\;dx^{n}\;dy^{n}+\int p_{X^{n},Y^{n}}\left(x^{n},y^{n}\right)\;\ln q_{Y^{n}|X^{n}}\left(y^{n}|x^{n}\right)\;dx^{n}\;dy^{n}. \end{array}$$

By using the logarithmic expectation in (15) and the following equality (see (55) and (56)):

(61) $$\begin{array}{c} \frac{p_{Y^{n}|X^{n}}\left(y^{n}|x^{n}\right)}{q_{Y^{n}|X^{n}}\left(y^{n}|x^{n}\right)}=\frac{1}{n}\sum_{i=1}^{n}\frac{r_{Y|X}\left(y_{i}|x_{i}\right)}{q_{Y|X}\left(y_{i}|x_{i}\right)}, \end{array}$$

we obtain (see Appendix D.1)

(62) $$\begin{array}{c} \int p_{X^{n},Y^{n}}\left(x^{n},y^{n}\right)\;\ln\left(\frac{p_{Y^{n}|X^{n}}\left(y^{n}|x^{n}\right)}{q_{Y^{n}|X^{n}}\left(y^{n}|x^{n}\right)}\right)\;dx^{n}\;dy^{n}=\int_{0}^{\infty }\frac{1}{u}\;\left[e^{-u}-f^{n-1}\left(\frac{u}{n}\right)\;g\left(\frac{u}{n}\right)\right]\;du, \end{array}$$

where, for u≥0,

(63) $$\begin{array}{c} f\left(u\right):=\int p_{X}\left(x\right)\;q_{Y|X}\left(y|x\right)\;\exp\left(-\frac{u\;r_{Y|X}\left(y|x\right)}{q_{Y|X}\left(y|x\right)}\right)\;dx\;dy, \end{array}$$

(64) $$\begin{array}{c} g\left(u\right):=\int p_{X}\left(x\right)\;r_{Y|X}\left(y|x\right)\;\exp\left(-\frac{u\;r_{Y|X}\left(y|x\right)}{q_{Y|X}\left(y|x\right)}\right)\;dx\;dy. \end{array}$$

Moreover, owing to the product form of $q_{n}$, it is shown in Appendix D.2 that

(65) $$\begin{array}{cc} \; & \;\int p_{X^{n},Y^{n}}\left(x^{n},y^{n}\right)\;\ln q_{Y^{n}|X^{n}}\left(y^{n}|x^{n}\right)\;dx^{n}\;dy^{n}\\ \; & =\int p_{X}\left(x\right)\;r_{Y|X}\left(y|x\right)\;\ln q_{Y|X}\left(y|x\right)\;dx\;dy+\left(n-1\right)\int p_{X}\left(x\right)\;q_{Y|X}\left(y|x\right)\;\ln q_{Y|X}\left(y|x\right)\;dx\;dy. \end{array}$$

Combining (60), (62), and (65), we express $h(Y^{n}|X^{n})$ as a double integral over X×Y, independently of *n* (rather than an integration over $X^{n}\times Y^{n}$):

(66) $$\begin{array}{cc} h(Y^{n}|X^{n}) & =\int_{0}^{\infty }\frac{1}{u}\;\left[f^{n-1}\left(\frac{u}{n}\right)\;g\left(\frac{u}{n}\right)-e^{-u}\right]\;du-\int p_{X}\left(x\right)\;r_{Y|X}\left(y|x\right)\;\ln q_{Y|X}\left(y|x\right)\;dx\;dy\\ \; & \;-\left(n-1\right)\int p_{X}\left(x\right)\;q_{Y|X}\left(y|x\right)\;\ln q_{Y|X}\left(y|x\right)\;dx\;dy. \end{array}$$

We next calculate the differential channel output entropy, $h(Y^{n})$, induced by $p_{Y^{n}|X^{n}}\left(\cdot |\cdot \right)$. From Appendix D.3,

(67) $$\begin{array}{c} p_{Y^{n}}\left(y^{n}\right)=\prod_{j=1}^{n}v\left(y_{j}\right)\cdot \frac{1}{n}\sum_{i=1}^{n}\frac{w(y_{i})}{v(y_{i})}, \end{array}$$

where, for all y∈Y,

(68) $$\begin{array}{c} v\left(y\right):=\int q_{Y|X}\left(y|x\right)\;p_{X}\left(x\right)\;dx, \end{array}$$

(69) $$\begin{array}{c} w\left(y\right):=\int r_{Y|X}\left(y|x\right)\;p_{X}\left(x\right)\;dx. \end{array}$$

By (1), the following identity holds for every positive random variable *Z* (see Appendix D.3):

(70) $$\begin{array}{c} E\left\{Z\ln Z\right\}=\int_{0}^{\infty }\frac{1}{u}\;\left[M_{Z}^{\prime }\left(0\right)\;e^{-u}-M_{Z}^{\prime }\left(-u\right)\right]\;du \end{array}$$

where $M_{Z}\left(u\right):=E\left\{e^{uZ}\right\}$. By setting $Z:=\frac{1}{n}\sum_{i=1}^{n}\frac{w(V_{i})}{v(V_{i})}$ where ${\left\{V_{i}\right\}}_{i=1}^{n}$ are i.i.d. random variables with the density function *v*, some algebraic manipulations give (see Appendix D.3)

(71) $$\begin{array}{cc} h(Y^{n}) & =\int_{0}^{\infty }\frac{1}{u}\left[t^{n-1}\left(\frac{u}{n}\right)\;s\left(\frac{u}{n}\right)-e^{-u}\right]\;du-\int w\left(y\right)\;\ln v\left(y\right)\;dy-\left(n-1\right)\int v\left(y\right)\ln v\left(y\right)\;dy, \end{array}$$

where

(72) $$\begin{array}{c} s\left(u\right):=\int w\left(y\right)\;\exp\left(-\frac{u\;w(y)}{v(y)}\right)\;dy,\;u\geq 0, \end{array}$$

(73) $$\begin{array}{c} t\left(u\right):=\int v\left(y\right)\;\exp\left(-\frac{u\;w(y)}{v(y)}\right)\;dy,\;u\geq 0. \end{array}$$

Combining (58), (66), and (71), we obtain the mutual information for the channel with jamming, which is given by

(74) $$\begin{array}{cc} I_{p}\left(X^{n};Y^{n}\right) & =\int_{0}^{\infty }\frac{1}{u}\left[t^{n-1}\left(\frac{u}{n}\right)\;s\left(\frac{u}{n}\right)-f^{n-1}\left(\frac{u}{n}\right)\;g\left(\frac{u}{n}\right)\right]\;du\\ \; & \;+\int p_{X}\left(x\right)\;r_{Y|X}\left(y|x\right)\;\ln q_{Y|X}\left(y|x\right)\;dx\;dy-\int w\left(y\right)\;\ln v\left(y\right)\;dy\\ \; & \;+\left(n-1\right)\left[\int p_{X}\left(x\right)\;q_{Y|X}\left(y|x\right)\;\ln q_{Y|X}\left(y|x\right)\;dx\;dy-\int v\left(y\right)\ln v\left(y\right)\;dy\right]. \end{array}$$

We next exemplify our results in the case where *q* is a binary symmetric channel (BSC) with crossover probability $\delta \in (0,\frac{1}{2})$ and *p* is a BSC with a larger crossover probability, $\varepsilon \in (\delta ,\frac{1}{2}]$. We assume that the input bits are i.i.d. and equiprobable. The specialization of our analysis to this setup is provided in Appendix D.4, showing that the mutual information of the channel $p_{X^{n},Y^{n}}$, fed by the binary symmetric source, is given by

(75) $$\begin{array}{cc} I_{p}\left(X^{n};Y^{n}\right) & =n\ln2-d\left(\varepsilon ∥\delta \right)-H_{b}\left(\varepsilon \right)-\left(n-1\right)H_{b}\left(\delta \right)\\ \; & \;+\int_{0}^{\infty }\{e^{-u}-{\left[\left(1-\delta \right)\;\exp\left(-\frac{(1-\varepsilon )u}{(1-\delta )n}\right)+\delta \;\exp\left(-\frac{\varepsilon u}{\delta n}\right)\right]}^{n-1}\\ \; & \;\cdot \left[\left(1-\varepsilon \right)\;\exp\left(-\frac{(1-\varepsilon )u}{(1-\delta )n}\right)+\varepsilon \;\exp\left(-\frac{\varepsilon u}{\delta n}\right)\right]\}\;\frac{du}{u}, \end{array}$$

where $H_{b}:\left[0,1\right]\to \left[0,\ln2\right]$ is the binary entropy function

(76) $$\begin{array}{c} H_{b}\left(x\right):=-x\ln\left(x\right)-\left(1-x\right)\ln\left(1-x\right),\;x\in \left[0,1\right], \end{array}$$

with the convention that 0ln0=0, and

(77) $$\begin{array}{c} d\left(\varepsilon ∥\delta \right):=\varepsilon \;\ln\left(\frac{\varepsilon }{\delta }\right)+\left(1-\varepsilon \right)\;\ln\left(\frac{1-\varepsilon }{1-\delta }\right),\;\left(\delta ,\varepsilon \right)\in {\left[0,1\right]}^{2} \end{array}$$

denotes the binary relative entropy. By the data processing inequality, the mutual information in (75) is smaller than that of the BSC with crossover probability δ:

(78) $$\begin{array}{cc} I_{q}\left(X^{n};Y^{n}\right) & =n\left(\ln2-H_{b}\left(\delta \right)\right). \end{array}$$

**Figure 3:**
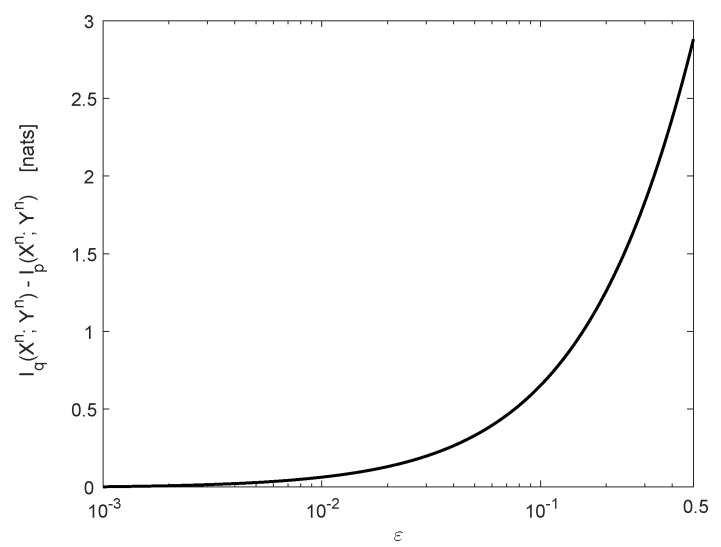
The degradation in mutual information for n=128. The jammer-free channel *q* is a binary symmetric channel (BSC) with crossover probability $\delta =10^{-3}$, and *r* is a BSC with crossover probability $\varepsilon \in \left(\delta ,\frac{1}{2}\right]$. The input bits are i.i.d. and equiprobable. The degradation in $I(X^{n};Y^{n})$ (nats) is displayed as a function of ε.

Figure 3 refers to the case where $\delta =10^{-3}$ and n=128. Here, $I_{q}\left(X^{n};Y^{n}\right)=87.71\;\mathrm{nats}$, and $I_{p}\left(X^{n};Y^{n}\right)$ is decreased by 2.88 nats due to the jammer (see Figure 3).

**Figure 4:**
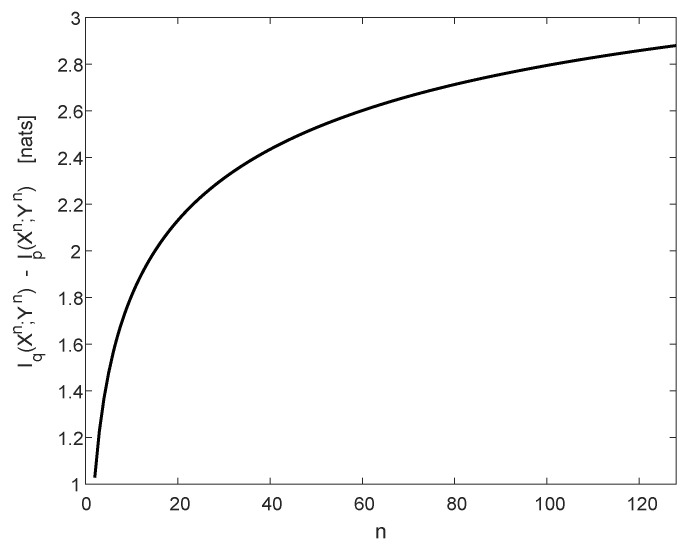
The degradation in mutual information as a function of *n*. The jammer-free channel $q_{Y|X}$ is a BSC with crossover probability $\delta =10^{-3}$, and $r_{Y|X}$ for the jammed symbol is a BSC with crossover probability $\varepsilon =\frac{1}{2}$. The input bits are i.i.d. and equiprobable.

Figure 4 refers to the case where $\delta =10^{-3}$ and $\varepsilon =\frac{1}{2}$ (referring to complete jamming of a single symbol, which is chosen uniformly at random), and it shows the difference in the mutual information $I(X^{n};Y^{n})$, as a function of the length *n*, between the jamming-free BSC with crossover probability δ and the channel with jamming.

To conclude, this subsection studies the change in the mutual information $I(X^{n};Y^{n})$ due to jamming, relative to the mutual information associated with the nominal channel without jamming. Due to the integral representations provided in our analysis, the calculation of the mutual information finally depends on one-dimensional integrals, as opposed to the original *n*-dimensional integrals, pertaining to the expressions that define the associated differential entropies.

## Appendix A. Proof of Theorem 1

Let ρ>0 be a non-integer real, and define the function $F_{\rho }:\left(0,\infty \right)\to R$ as follows:

(A1) $$F_{\rho }\left(\mu \right):=\int_{0}^{\infty }\frac{1}{u^{\rho +1}}\;\left(e^{-\mu u}-\sum_{j=0}^{\left\lfloor \rho \right\rfloor }\left\{\frac{{\left(-1\right)}^{j}}{j!}\;{\left(\mu -1\right)}^{j}u^{j}\right\}\;e^{-u}\right)\;du,\;\mu >0,$$

with the convention that $0^{0}:=\lim_{x\to 0^{+}}x^{x}=1$. By the Taylor series expansion of $e^{-\mu u}$ as a function of μ around μ=1, we find that for small positive *u*, the integrand of (A1) scales as $u^{-(\rho -\lfloor \rho \rfloor )}$ with ρ−⌊ρ⌋∈(0,1). Furthermore, for large *u*, the same integrand scales as $u^{-(\rho +1)}e^{-\min\{\mu ,1\}u}$. This guarantees the convergence of the integral, and so, $F_{\rho }\left(\cdot \right)$ is well defined and finite in the interval (0,∞).

From (A1), $F_{\rho }\left(1\right)=0$ (for μ=1, the integrand of (A1) is identically zero on (0,∞)). Differentiation *ℓ* times with respect to μ, under the integration sign with $\ell \in \left\{0,\ldots ,\lfloor \rho \rfloor \right\}$, gives according to Leibniz integral rule

(A2) $$\begin{array}{c} F_{\rho }^{\left(\ell \right)}\left(\mu \right)=\int_{0}^{\infty }\frac{1}{u^{\rho +1}}\left[{\left(-1\right)}^{\ell }u^{\ell }e^{-\mu u}-\sum_{j=\ell }^{\left\lfloor \rho \right\rfloor }\left\{\frac{{\left(-1\right)}^{j}}{(j-\ell )!}\cdot {\left(\mu -1\right)}^{j-\ell }u^{j}\right\}\;e^{-u}\right]\;du, \end{array}$$

which implies that

(A3) $$\begin{array}{c} F_{\rho }^{\left(\ell \right)}\left(1\right)=0,\;\ell =0,\ldots ,\left\lfloor \rho \right\rfloor . \end{array}$$

We next calculate $F_{\rho }^{\left(k\right)}\left(\mu \right)$ for k:=⌊ρ⌋+1 and μ>0. By invoking the Leibniz integral rule,

(A4) $$\begin{array}{cc} F_{\rho }^{\left(k\right)}\left(\mu \right) & =\int_{0}^{\infty }\frac{1}{u^{\rho +1}}\;\frac{\partial^{k}}{\partial \mu^{k}}\left\{e^{-\mu u}-\sum_{j=0}^{\left\lfloor \rho \right\rfloor }\left\{\frac{{\left(-1\right)}^{j}}{j!}\cdot {\left(\mu -1\right)}^{j}u^{j}\right\}\;e^{-u}\right\}\;du\\ \; & =\int_{0}^{\infty }\frac{{\left(-u\right)}^{k}e^{-\mu u}}{u^{\rho +1}}\;du\\ \; & ={\left(-1\right)}^{k}\int_{0}^{\infty }u^{k-\rho -1}e^{-\mu u}\;du\\ \; & ={\left(-1\right)}^{k}\int_{0}^{\infty }{\left(\frac{t}{\mu }\right)}^{k-\rho -1}\;e^{-t}\;\mu^{-1}\;dt\\ \; & ={\left(-1\right)}^{k}\mu^{\rho -k}\;\Gamma \left(k-\rho \right). \end{array}$$

Hence, from (A3) and (A4),

(A5) $$\begin{array}{ll} \; & F_{\rho }\left(1\right)=\ldots =F_{\rho }^{\left(\lfloor \rho \rfloor \right)}\left(1\right)=0, \end{array}$$

(A6) $$\begin{array}{ll} \; & F_{\rho }^{\left(k\right)}\left(\mu \right)={\left(-1\right)}^{k}\mu^{\rho -k}\;\Gamma \left(k-\rho \right),\;k:=\left\lfloor \rho \right\rfloor +1,\;\mu >0. \end{array}$$

By integrating both sides of (A6) with respect to μ, successively *k* times, the equalities in (A5) imply that

(A7) $$\begin{array}{c} F_{\rho }\left(\mu \right)=\frac{{\left(-1\right)}^{k}\;\Gamma \left(k-\rho \right)\;\mu^{\rho }}{\prod_{i=0}^{k-1}\left(\rho -i\right)}+\sum_{i=0}^{k-1}c_{i}\left(\rho \right)\;{\left(\mu -1\right)}^{i},\;k:=\left\lfloor \rho \right\rfloor +1,\;\mu >0, \end{array}$$

with some suitable constants ${\left\{c_{i}\left(\rho \right)\right\}}_{i=0}^{k-1}$. Since $F_{\rho }\left(1\right)=0$ (see (A5)), (A7) implies that

(A8) $$\begin{array}{c} c_{0}\left(\rho \right)=\frac{{\left(-1\right)}^{k+1}\;\Gamma \left(k-\rho \right)}{\prod_{i=0}^{k-1}\left(\rho -i\right)}, \end{array}$$

and since (by assumption) ρ is a non-integer, the denominator on the right-hand side of (A8) is nonzero. Moreover, since $F_{\rho }^{\left(\ell \right)}\left(1\right)=0$ for all ℓ∈{1,…k−1} (see (A5)), differentiation of both sides of (A7) *ℓ* times at μ=1 yields

(A9) $$\begin{array}{c} c_{\ell }\left(\rho \right):=\frac{{\left(-1\right)}^{k+1}\;\Gamma \left(k-\rho \right)\;\prod_{i=0}^{\ell -1}\left(\rho -i\right)}{\ell !\;\prod_{i=0}^{k-1}\left(\rho -i\right)},\;\ell =1,\ldots ,k-1. \end{array}$$

Substituting (A8) and (A9) into (A7) gives

(A10) $$\begin{array}{c} F_{\rho }\left(\mu \right)=\frac{{\left(-1\right)}^{k}\;\Gamma \left(k-\rho \right)}{\prod_{i=0}^{k-1}\left(\rho -i\right)}\left[\mu^{\rho }-1-\sum_{\ell =1}^{k-1}\left\{\;\frac{1}{\ell !}\;\prod_{i=0}^{\ell -1}\left(\rho -i\right)\;{\left(\mu -1\right)}^{\ell }\;\right\}\right],\;\mu >0. \end{array}$$

Combining (A1) with (A10) and rearranging the terms, we obtain

(A11) $$\begin{array}{cc} \mu^{\rho } & =1+\sum_{\ell =1}^{k-1}\left\{\frac{1}{\ell !}\;\prod_{i=0}^{\ell -1}\left(\rho -i\right)\;{\left(\mu -1\right)}^{\ell }\;\right\}\\ \; & \;+\frac{{\left(-1\right)}^{k-1}\;\prod_{i=0}^{k-1}\left(\rho -i\right)}{\Gamma (k-\rho )}\int_{0}^{\infty }\frac{1}{u^{\rho +1}}\;\left(\;\sum_{j=0}^{\left\lfloor \rho \right\rfloor }\left\{\frac{{\left(-1\right)}^{j}}{j!}\;{\left(\mu -1\right)}^{j}u^{j}\right\}\;e^{-u}-e^{-\mu u}\right)\;du. \end{array}$$

Setting μ:=X≥0 and taking expectations of both sides of (A11) imply, by swapping the order of integration and expectation (which is validated due to Fubini’s theorem), that the following equality holds (see (4) and (10)):

(A12) $$\begin{array}{cc} E\left\{X^{\rho }\right\} & =1+\sum_{\ell =1}^{k-1}\left\{\;\frac{1}{\ell !}\;\prod_{i=0}^{\ell -1}\left(\rho -i\right)\;\alpha_{\ell }\;\right\}\\ \; & \;+\frac{{\left(-1\right)}^{k-1}\;\prod_{i=0}^{k-1}\left(\rho -i\right)}{\Gamma (k-\rho )}\int_{0}^{\infty }\frac{1}{u^{\rho +1}}\;\left(\;\sum_{j=0}^{\left\lfloor \rho \right\rfloor }\left\{\frac{{\left(-1\right)}^{j}\;\alpha_{j}}{j!}\;u^{j}\right\}\;e^{-u}-M_{X}\left(-u\right)\right)\;du. \end{array}$$

We next rewrite and simplify both terms in the right side of (A12) as follows:

(A13) $$\begin{array}{cc} 1+\sum_{\ell =1}^{k-1}\left\{\;\frac{1}{\ell !}\;\prod_{i=0}^{\ell -1}\left(\rho -i\right)\;\alpha_{\ell }\;\right\} & =1+\sum_{\ell =1}^{k-1}\left\{\;\frac{1}{\Gamma (\ell +1)}\;\frac{\Gamma (\rho +1)}{\Gamma (\rho -\ell +1)}\cdot \alpha_{\ell }\;\right\} \end{array}$$

(A14) $$\begin{array}{cc} \; & \;=1+\frac{1}{1+\rho }\;\sum_{\ell =1}^{k-1}\left\{\;\frac{1}{\Gamma (\ell +1)}\;\frac{\Gamma (\rho +2)}{\Gamma (\rho -\ell +1)}\cdot \alpha_{\ell }\;\right\} \end{array}$$

(A15) $$\begin{array}{cc} \; & \;=1+\frac{1}{1+\rho }\;\sum_{\ell =1}^{k-1}\frac{\alpha_{\ell }}{B(\ell +1,\rho -\ell +1)} \end{array}$$

(A16) $$\begin{array}{cc} \; & \;=\frac{1}{1+\rho }\;\sum_{\ell =0}^{k-1}\frac{\alpha_{\ell }}{B(\ell +1,\rho -\ell +1)}, \end{array}$$

and

(A17) $$\begin{array}{cc} \frac{{\left(-1\right)}^{k-1}\;\prod_{i=0}^{k-1}\left(\rho -i\right)}{\Gamma (k-\rho )} & =\frac{{\left(-1\right)}^{k-1}\;\Gamma \left(\rho +1\right)}{\Gamma (k-\rho )\;\Gamma (\rho -k+1)} \end{array}$$

(A18) $$\begin{array}{cc} \; & \;={\left(-1\right)}^{k-1}\;\Gamma \left(\rho +1\right)\cdot \frac{\sin\left(\pi (k-\rho )\right)}{\pi } \end{array}$$

(A19) $$\begin{array}{cc} \; & \;=\frac{\rho \;\sin(\pi \rho )\;\Gamma (\rho )}{\pi }. \end{array}$$

Equations (A13), (A14), (A17), and (A19) are based on the recursion (see, e.g., [26] (Identity (8.331)))

(A20) $$\begin{array}{c} \Gamma (x+1)=x\;\Gamma (x),\;x>0, \end{array}$$

(A15) relies on the relation between the Beta and Gamma functions in (6); (A16) is based on the following equality (see (6), (A20), and recall that Γ(1)=1):

(A21) $$\begin{array}{c} B\left(1,\rho +1\right)=\frac{\Gamma (1)\;\Gamma (\rho +1)}{\Gamma (\rho +2)}=\frac{1}{\rho +1}, \end{array}$$

and finally, (A19) holds by using the identity (see, e.g., [26] (page 905, Identity (8.334)))

(A22) $$\begin{array}{cc} \; & \Gamma \left(x\right)\;\Gamma \left(1-x\right)=\frac{\pi }{\sin(\pi x)},\;\forall \;x\in \left(0,1\right), \end{array}$$

with x:=k−ρ=⌊ρ⌋+1−ρ∈(0,1) (since, by assumption, ρ is a non-integer). Combining (A12)–(A19) gives (9) (recall that $\alpha_{0}:=1$, and k−1:=⌊ρ⌋ holds by (A6)).

We finally prove (11). By (10), for all j∈N,

(A23) αj=E(X−1)j=∑ℓ=0j(−1)j−ℓjℓEXℓ=∑ℓ=0j(−1)j−ℓΓ(j+1)MX(ℓ)(0)Γ(ℓ+1)Γ(j−ℓ+1)=1j+1∑ℓ=0j(−1)j−ℓΓ(j+2)MX(ℓ)(0)Γ(ℓ+1)Γ(j−ℓ+1)=1j+1∑ℓ=0j(−1)j−ℓMX(ℓ)(0)B(ℓ+1,j−ℓ+1).

## Appendix B. Complementary Details of the Analysis in Section 3.2

### Appendix B.1. Proof of Equation (36)

For all u>0,

(A24) $$\begin{array}{cc} M_{D_{n}}\left(-u\right) & =E\left\{\exp\left(-u{\left({\hat{\theta }}_{n}-\theta \right)}^{2}\right)\right\} \end{array}$$

(A25) $$\begin{array}{cc} \; & \;=E\left\{\frac{1}{2\sqrt{\pi u}}\int_{-\infty }^{\infty }e^{j\omega ({\hat{\theta }}_{n}-\theta )}\;e^{-\omega^{2}/\left(4u\right)}\;d\omega \right\} \end{array}$$

(A26) $$\begin{array}{cc} \; & \;=\frac{1}{2\sqrt{\pi u}}\int_{-\infty }^{\infty }e^{-j\omega \theta }\;E\{e^{j\omega {\hat{\theta }}_{n}}\}\;e^{-\omega^{2}/\left(4u\right)}\;d\omega \end{array}$$

(A27) $$\begin{array}{cc} \; & \;=\frac{1}{2\sqrt{\pi u}}\int_{-\infty }^{\infty }e^{-j\omega \theta }\;E\left\{\exp\left(\frac{j\omega }{n}\;\sum_{i=1}^{n}X_{i}\right)\right\}\;e^{-\omega^{2}/\left(4u\right)}\;d\omega \end{array}$$

(A28) $$\begin{array}{cc} \; & \;=\frac{1}{2\sqrt{\pi u}}\int_{-\infty }^{\infty }e^{-j\omega \theta }\;\phi_{X}^{n}\left(\frac{\omega }{n}\right)\;e^{-\omega^{2}/\left(4u\right)}\;d\omega , \end{array}$$

where (A24) is (31); (A25) relies on (35); (A26) holds by interchanging expectation and integration (due to Fubini’s theorem); (A27) is due to (28), and (A28) holds by the assumption that $X_{1},\ldots ,X_{n}$ are i.i.d.

### Appendix B.2. Derivation of the Upper Bound in (40)

For all ρ>0,

(A29) $$\begin{array}{cc} E\{{\left|{\hat{\theta }}_{n}-\theta \right|}^{\rho }\} & =\int_{0}^{\infty }P({\left|{\hat{\theta }}_{n}-\theta \right|}^{\rho }\geq t)\;dt\\ \; & =\int_{0}^{\infty }P({\left|{\hat{\theta }}_{n}-\theta \right|}^{\rho }\geq \varepsilon^{\rho })\;\rho \;\varepsilon^{\rho -1}\;d\varepsilon \\ \; & =\int_{0}^{\infty }P(\left|{\hat{\theta }}_{n}-\theta \right|\geq \varepsilon )\;\rho \;\varepsilon^{\rho -1}\;d\varepsilon . \end{array}$$

We next use the Chernoff bound for upper bounding $P(\left|{\hat{\theta }}_{n}-\theta \right|\geq \varepsilon )$ for all ε>0,

(A30) $$\begin{array}{cc} P({\hat{\theta }}_{n}-\theta \geq \varepsilon ) & =P\left(\sum_{i=1}^{n}\left(X_{i}-\theta \right)\geq n\varepsilon \right)\\ \; & \leq \underset{s\geq 0}{\inf}\;\left\{e^{-sn\varepsilon }\;E\left\{\exp\left(s\sum_{i=1}^{n}\left(X_{i}-\theta \right)\right)\right\}\right\}\\ \; & =\underset{s\geq 0}{\inf}\;\left\{e^{-sn\varepsilon }\;\prod_{i=1}^{n}E\left\{e^{s(X_{i}-\theta )}\right\}\right\}\\ \; & =\underset{s\geq 0}{\inf}\;\left\{e^{-sn\varepsilon }\;{\left(\theta \;e^{s(1-\theta )}+\left(1-\theta \right)\;e^{-s\theta }\right)}^{n}\right\}\\ \; & =\underset{s\geq 0}{\inf}\;\left\{e^{-ns\varepsilon +nH_{\theta }\left(s\right)}\right\} \end{array}$$

with θ∈[0,1], and

(A31) $$\begin{array}{c} H_{\theta }\left(s\right):=\ln\left(\theta \;e^{s(1-\theta )}+\left(1-\theta \right)\;e^{-s\theta }\right),\;s\geq 0. \end{array}$$

We now use an upper bound on $H_{\theta }\left(s\right)$ for every s≥0. By Theorem 3.2 and Lemma 3.3 in [39] (see also Lemma 2.4.6 in [40]), we have

(A32) $$\begin{array}{c} H_{\theta }\left(s\right)\leq C\left(\theta \right)\;s^{2} \end{array}$$

with

(A33) $$C\left(\theta \right):=\{\begin{array}{cc} 0,\; & \mathrm{if}\;\theta =0,\\ \frac{1-2\theta }{4\ln\left(\frac{1-\theta }{\theta }\right)}, & \mathrm{if}\;\theta \in \left(0,\frac{1}{2}\right),\\ \frac{1}{2}\;\theta \left(1-\theta \right), & \mathrm{if}\;\theta \in \left[\frac{1}{2},1\right]. \end{array}$$

Combining (A30) and (A32) yields

(A34) $$\begin{array}{cc} P({\hat{\theta }}_{n}-\theta \geq \varepsilon ) & \leq \underset{s\geq 0}{\inf}\;\left\{e^{-n\varepsilon s+nC\left(\theta \right)s^{2}}\right\}\\ \; & =\exp\left(-\frac{n\varepsilon^{2}}{4C(\theta )}\right). \end{array}$$

Similarly, it is easy to show that the same Chernoff bound applies also to $P({\hat{\theta }}_{n}-\theta \leq -\varepsilon )$, which overall gives

(A35) $$\begin{array}{c} P(\left|{\hat{\theta }}_{n}-\theta \right|\geq \varepsilon )\leq 2\exp\left(-\frac{n\varepsilon^{2}}{4C(\theta )}\right). \end{array}$$

Inequality (A35) is a refined version of Hoeffding’s inequality (see Section 2.4.4 in [40]), which is derived for the Bernoulli distribution (see (A30)) and by invoking the Chernoff bound; moreover, (A35) coincides with Hoeffding’s inequality in the special case $\theta =\frac{1}{2}$ (which, from (A33), yields $C\left(\theta \right)=\frac{1}{8}$). In view of the fact that (A35) forms a specialization of Theorem 2.4.7 in [40], it follows that the Bernoulli case is the worst one (in the sense of leading to the looser upper bound) among all probability distributions whose support is the interval [0,1] and whose expected value is θ∈[0,1]. However, in the Bernoulli case, a simple symmetry argument applies for improving the bound (A35) as follows. Since $\left\{X_{i}\right\}$ are i.i.d., Bernoulli with mean θ, then obviously, $\left\{1-X_{i}\right\}$ are Bernoulli, i.i.d. with mean 1−θ and (from (28))

(A36) $$\begin{array}{c} {\hat{\theta }}_{n}\left(1-X_{1},\ldots ,1-X_{n}\right)=1-{\hat{\theta }}_{n}\left(X_{1},\ldots ,X_{n}\right), \end{array}$$

which implies that the error estimation is identical in both cases. Hence, $P(|{\hat{\theta }}_{n}-\theta |\geq \varepsilon )$ is symmetric around $\theta =\frac{1}{2}$. It can be verified that

(A37) $$\begin{array}{c} \min\left\{C(\theta ),\;C(1-\theta )\right\}=\frac{1}{2}\;\theta \left(1-\theta \right),\;\forall \;\theta \in \left[0,1\right], \end{array}$$

which follows from (A33) and since C(θ)>C(1−θ) for all $\theta \in (0,\frac{1}{2})$ (see Figure 2.1 in [40]). In view of (A37) and the above symmetry consideration, the upper bound in (A35) is improved for values of $\theta \in (0,\frac{1}{2})$, which therefore gives

(A38) $$\begin{array}{c} P)|{\hat{\theta }}_{n}-\theta |\geq \varepsilon )\leq 2\exp\left(-\frac{n\varepsilon^{2}}{2\theta (1-\theta )}\right),\;\forall \;\theta \in \left[0,1\right],\;\varepsilon >0. \end{array}$$

From (28), the probability in (A38) vanishes if θ=0 or θ=1. Consequently, for ρ>0,

(A39) $$\begin{array}{cc} E\left\{{\left|{\hat{\theta }}_{n}-\theta \right|}^{\rho }\right\} & =\int_{0}^{\infty }P\left(\left|{\hat{\theta }}_{n}-\theta \right|\geq \varepsilon \right)\;\rho \;\varepsilon^{\rho -1}\;d\varepsilon \end{array}$$

(A40) $$\begin{array}{cc} \; & \;\leq \int_{0}^{\infty }2\exp\left(-\frac{n\varepsilon^{2}}{2\theta (1-\theta )}\right)\;\rho \;\varepsilon^{\rho -1}\;d\varepsilon \end{array}$$

(A41) $$\begin{array}{cc} \; & \;=\rho \;{\left(2\theta (1-\theta )\right)}^{\rho /2}\int_{0}^{\infty }u^{\rho /2-1}\;e^{-u}\;du\cdot n^{-\rho /2} \end{array}$$

(A42) $$\begin{array}{cc} \; & \;=\rho \;\Gamma \left(\frac{\rho }{2}\right){\left(2\theta (1-\theta )\right)}^{\rho /2}\cdot n^{-\rho /2} \end{array}$$

(A43) $$\begin{array}{c} =K\left(\rho ,\theta \right)\cdot n^{-\rho /2}, \end{array}$$

where (A39)–(A43) hold, respectively, due to (A29), (A38), the substitution $u:=\frac{n\varepsilon^{2}}{2\theta (1-\theta )}$, (7), and (41).

## Appendix C. Complementary Details of the Analysis in Section 3.3

We start by proving (50). In view of (48), for α∈(0,1)∪(1,∞),

(A44) $$\begin{array}{c} h_{\alpha }\left(X^{n}\right)=\frac{1}{1-\alpha }\;\log E\left[f^{\alpha -1}\left(X^{n}\right)\right], \end{array}$$

where $X^{n}:=\left(X_{1},\ldots ,X_{n}\right)$. For α>1, we get

(A45) $$\begin{array}{cc} E\left[f^{\alpha -1}\left(X^{n}\right)\right] & =C_{n}^{\alpha -1}\;E\left\{{\left[1+\sum_{i=1}^{n}g\left(X_{i}\right)\right]}^{q(1-\alpha )}\right\} \end{array}$$

(A46) $$\begin{array}{cc} \; & \;=C_{n}^{\alpha -1}\;\int_{R^{n}}f\left(x^{n}\right)\cdot \frac{1}{\Gamma \left(q(\alpha -1)\right)}\int_{0}^{\infty }t^{q(\alpha -1)-1}\exp\left\{-\left(1+\sum_{i=1}^{n}g\left(x_{i}\right)\right)t\right\}\;dt \end{array}$$

(A47) $$\begin{array}{cc} \; & \;=\frac{C_{n}^{\alpha -1}}{\Gamma \left(q(\alpha -1)\right)}\int_{0}^{\infty }t^{q(\alpha -1)-1}\;e^{-t}\;E\left[\exp\left(-t\sum_{i=1}^{n}g\left(X_{i}\right)\right)\right]\;dt. \end{array}$$

where (A45) holds due to (42); (A46) follows from (49), and (A47) holds by swapping the order of integrations, which is validated by Fubini’s theorem. Furthermore, from (42) and (49),

(A48) $$\begin{array}{cc} f(x^{n}) & =\frac{C_{n}}{{\left(1+\sum_{i=1}^{n}g\left(x_{i}\right)\right)}^{q}}\\ \; & =\frac{C_{n}}{\Gamma (q)}\int_{0}^{\infty }u^{q-1}e^{-u}\exp\left(-u\sum_{i=1}^{n}g\left(x_{i}\right)\right)du,\;\forall \;x^{n}\in R^{n}, \end{array}$$

and it follows from (A48) and by swapping the order of integrations (due to Fubini’s theorem),

(A49) $$\begin{array}{cc} E\left[\exp\left(-t\sum_{i=1}^{n}g\left(X_{i}\right)\right)\right] & =\frac{C_{n}}{\Gamma (q)}\int_{0}^{\infty }u^{q-1}e^{-u}\int_{R^{n}}\exp\left(-\left(t+u\right)\sum_{i=1}^{n}g\left(x_{i}\right)\right)dx^{n}\;du\\ \; & =\frac{C_{n}}{\Gamma (q)}\int_{0}^{\infty }u^{q-1}e^{-u}\left\{\prod_{i=1}^{n}\int_{-\infty }^{\infty }\exp\left(-\left(t+u\right)g\left(x_{i}\right)\right)\;dx_{i}\right\}du\\ \; & =\frac{C_{n}}{\Gamma (q)}\int_{0}^{\infty }u^{q-1}e^{-u}{\left(\int_{-\infty }^{\infty }\exp\left(-(t+u)\;g(x)\right)\;dx\right)}^{n}\;du\\ \; & =\frac{C_{n}}{\Gamma (q)}\int_{0}^{\infty }u^{q-1}e^{-u}Z^{n}\left(t+u\right)\;du, \end{array}$$

where (A49) holds by the definition of Z(·) in (44). Finally, combining (45), (A44), (A47), and (A49) gives (50).

The proof of (51)–(54) is a straightforward calculation, which follows by combining (A44), (A45), (A49) and Theorem 1 (we replace $\left\{\alpha_{j}\right\}$ in Theorem 1 with $\left\{\beta_{j}\left(n\right)\right\}$ in order not to confuse the order α of the Rényi entropy of $X^{n}$).

## Appendix D. Calculations of the n-Dimensional Integrals in Section 3.4

### Appendix D.1. Proof of Equations (62)–(64)

$$\begin{array}{cc} \; & \;\int p_{X^{n},Y^{n}}\left(x^{n},y^{n}\right)\;\ln\left(\frac{p_{Y^{n}|X^{n}}\left(y^{n}|x^{n}\right)}{q_{Y^{n}|X^{n}}\left(y^{n}|x^{n}\right)}\right)\;dx^{n}\;dy^{n}\\ \; & =\int p_{X^{n},Y^{n}}\left(x^{n},y^{n}\right)\;\ln\left(\frac{1}{n}\sum_{i=1}^{n}\frac{r_{Y|X}\left(y_{i}|x_{i}\right)}{q_{Y|X}\left(y_{i}|x_{i}\right)}\right)\;dx^{n}\;dy^{n} \end{array}$$

(A50) $$\begin{array}{cc} \; & \;=\int_{0}^{\infty }\frac{1}{u}\;\left[e^{-u}-\int p_{X^{n},Y^{n}}\left(x^{n},y^{n}\right)\exp\left(-\frac{u}{n}\sum_{i=1}^{n}\frac{r_{Y|X}\left(y_{i}|x_{i}\right)}{q_{Y|X}\left(y_{i}|x_{i}\right)}\right)\;dx^{n}\;dy^{n}\right]\;du \end{array}$$

$$\;=\int_{0}^{\infty }\frac{1}{u}\;[e^{-u}-\int \frac{1}{n}\sum_{i=1}^{n}\left\{\prod_{j\neq i}q_{Y|X}\left(y_{j}|x_{j}\right)\;p_{X}\left(x_{j}\right)\cdot r_{Y|X}\left(y_{i}|x_{i}\right)\;p_{X}\left(x_{i}\right)\right\}$$

(A51) $$\begin{array}{cc} \; & \;\cdot \exp\left(-\frac{u}{n}\sum_{i=1}^{n}\frac{r_{Y|X}\left(y_{i}|x_{i}\right)}{q_{Y|X}\left(y_{i}|x_{i}\right)}\right)\;dx^{n}\;dy^{n}]\;du \end{array}$$

$$\;=\int_{0}^{\infty }\frac{1}{u}\;[e^{-u}-\int \frac{1}{n}\sum_{i=1}^{n}\{\prod_{j\neq i}q_{Y|X}\left(y_{j}|x_{j}\right)\;p_{X}\left(x_{j}\right)\;\exp\left(-\frac{u}{n}\;\frac{r_{Y|X}\left(y_{j}|x_{j}\right)}{q_{Y|X}\left(y_{j}|x_{j}\right)}\right)$$

(A52) $$\begin{array}{cc} \; & \;\cdot r_{Y|X}\left(y_{i}|x_{i}\right)\;p_{X}\left(x_{i}\right)\exp\left(-\frac{u}{n}\;\frac{r_{Y|X}\left(y_{i}|x_{i}\right)}{q_{Y|X}\left(y_{i}|x_{i}\right)}\right)\}\;dx^{n}\;dy^{n}]\;du \end{array}$$

$$\;=\int_{0}^{\infty }\frac{1}{u}\;[e^{-u}-\frac{1}{n}\sum_{i=1}^{n}\{\prod_{j\neq i}\int q_{Y|X}\left(y_{j}|x_{j}\right)\;p_{X}\left(x_{j}\right)\;\exp\left(-\frac{u}{n}\;\frac{r_{Y|X}\left(y_{j}|x_{j}\right)}{q_{Y|X}\left(y_{j}|x_{j}\right)}\right)\;dx_{j}\;dy_{j}$$

(A53) $$\begin{array}{cc} \; & \;\cdot \int r_{Y|X}\left(y_{i}|x_{i}\right)\;p_{X}\left(x_{i}\right)\exp\left(-\frac{u}{n}\;\frac{r_{Y|X}\left(y_{i}|x_{i}\right)}{q_{Y|X}\left(y_{i}|x_{i}\right)}\right)\;dx_{i}\;dy_{i}\;\}]\;du \end{array}$$

$$\;=\int_{0}^{\infty }\frac{1}{u}\;[e^{-u}-\frac{1}{n}\sum_{i=1}^{n}\{{\left(\int q_{Y|X}\left(y|x\right)\;p_{X}\left(x\right)\;\exp\left(-\frac{u}{n}\;\frac{r_{Y|X}\left(y|x\right)}{q_{Y|X}\left(y|x\right)}\right)\;dx\;dy\right)}^{n-1}$$

(A54) $$\begin{array}{cc} \; & \;\cdot \int r_{Y|X}\left(y|x\right)\;p_{X}\left(x\right)\exp\left(-\frac{u}{n}\;\frac{r_{Y|X}\left(y|x\right)}{q_{Y|X}\left(y|x\right)}\right)\;dx\;dy\;\}]\;du \end{array}$$

$$\;=\int_{0}^{\infty }\frac{1}{u}\;[e^{-u}-{\left(\int q_{Y|X}\left(y|x\right)\;p_{X}\left(x\right)\;\exp\left(-\frac{u}{n}\;\frac{r_{Y|X}\left(y|x\right)}{q_{Y|X}\left(y|x\right)}\right)\;dx\;dy\right)}^{n-1}$$

(A55) $$\begin{array}{cc} \; & \;\cdot \int r_{Y|X}\left(y|x\right)\;p_{X}\left(x\right)\exp\left(-\frac{u}{n}\;\frac{r_{Y|X}\left(y|x\right)}{q_{Y|X}\left(y|x\right)}\right)\;dx\;dy\;]\;du \end{array}$$

(A56) $$\begin{array}{c} =\int_{0}^{\infty }\frac{1}{u}\;\left[e^{-u}-f^{n-1}\left(\frac{u}{n}\right)\;g\left(\frac{u}{n}\right)\right]\;du, \end{array}$$

where f(·) and g(·) are defined in (63) and (64), respectively. Consequently, f(0)=g(0)=1, and 0≤f(u),g(u)≤1 for all u>0.

### Appendix D.2. Proof of Equation (65)

$$\begin{array}{c} \;\int p_{X^{n},Y^{n}}\left(x^{n},y^{n}\right)\;\ln q_{Y^{n}|X^{n}}\left(y^{n}|x^{n}\right)\;dx^{n}\;dy^{n} \end{array}$$

(A57) $$\begin{array}{c} \;=\int p_{X^{n},Y^{n}}\left(x^{n},y^{n}\right)\;\sum_{j=1}^{n}\ln q_{Y|X}\left(y_{j}|x_{j}\right)\;dx^{n}\;dy^{n} \end{array}$$

(A58) $$\begin{array}{c} \;=\int \prod_{\ell =1}^{n}p_{X}\left(x_{\ell }\right)\cdot \frac{1}{n}\sum_{i=1}^{n}\left\{\prod_{\ell \neq i}q_{Y|X}\left(y_{\ell }|x_{\ell }\right)\;r_{Y|X}\left(y_{i}|x_{i}\right)\right\}\sum_{j=1}^{n}\ln q_{Y|X}\left(y_{j}|x_{j}\right)\;dx^{n}\;dy^{n} \end{array}$$

(A59) $$\begin{array}{c} \;=\int \prod_{\ell =1}^{n}p_{X}\left(x_{\ell }\right)\cdot \frac{1}{n}\sum_{i=1}^{n}\sum_{j=1}^{n}\left\{\prod_{\ell \neq i}q_{Y|X}\left(y_{\ell }|x_{\ell }\right)\cdot r_{Y|X}\left(y_{i}|x_{i}\right)\;\ln q_{Y|X}\left(y_{j}|x_{j}\right)\right\}\;dx^{n}\;dy^{n} \end{array}$$

(A60) $$\begin{array}{c} \;=\frac{1}{n}\;\int_{X^{n}}\prod_{\ell =1}^{n}p_{X}\left(x_{\ell }\right)\;\left(\sum_{i=1}^{n}\sum_{j=1}^{n}\int_{Y^{n}}\prod_{\ell \neq i}q_{Y|X}\left(y_{\ell }|x_{\ell }\right)\cdot r_{Y|X}\left(y_{i}|x_{i}\right)\;\ln q_{Y|X}\left(y_{j}|x_{j}\right)\;dy^{n}\right)\;dx^{n}. \end{array}$$

We next calculate the inner integral on the right-hand side of (A60). For i=j,

(A61) $$\begin{array}{cc} \; & \;\int_{Y^{n}}\prod_{\ell \neq i}q_{Y|X}\left(y_{\ell }|x_{\ell }\right)\cdot r_{Y|X}\left(y_{i}|x_{i}\right)\;\ln q_{Y|X}\left(y_{j}|x_{j}\right)\;dy^{n}\\ \; & =\prod_{\ell \neq i}\int_{Y}q_{Y|X}\left(y_{\ell }|x_{\ell }\right)\;dy_{\ell }\cdot \int_{Y}r_{Y|X}\left(y_{i}|x_{i}\right)\;\ln q_{Y|X}\left(y_{i}|x_{i}\right)\;dy_{i}\\ \; & =\int_{Y}r_{Y|X}\left(y|x_{i}\right)\;\ln q_{Y|X}\left(y|x_{i}\right)\;dy, \end{array}$$

else, if i≠j,

(A62) $$\begin{array}{cc} \; & \;\int_{Y^{n}}\prod_{\ell \neq i}q_{Y|X}\left(y_{\ell }|x_{\ell }\right)\cdot r_{Y|X}\left(y_{i}|x_{i}\right)\;\ln q_{Y|X}\left(y_{j}|x_{j}\right)\;dy^{n}\\ \; & =\prod_{\ell \notin \{i,j\}}\int_{Y}q_{Y|X}\left(y_{\ell }|x_{\ell }\right)\;dy_{\ell }\cdot \int_{Y}q_{Y|X}\left(y_{j}|x_{j}\right)\;\ln q_{Y|X}\left(y_{j}|x_{j}\right)\;dy_{j}\cdot \int_{Y}r_{Y|X}\left(y_{i}|x_{i}\right)\;dy_{i}\\ \; & =\int_{Y}q_{Y|X}\left(y|x_{j}\right)\;\ln q_{Y|X}\left(y|x_{j}\right)\;dy. \end{array}$$

Hence, from (A60)–(A62),

(A63) $$\begin{array}{cc} \; & \int p_{X^{n},Y^{n}}\left(x^{n},y^{n}\right)\;\ln q_{Y^{n}|X^{n}}\left(y^{n}|x^{n}\right)\;dx^{n}\;dy^{n}\\ \; & =\frac{1}{n}\;\int_{X^{n}}\prod_{\ell =1}^{n}p_{X}\left(x_{\ell }\right)\;\left(\sum_{i=1}^{n}\int_{Y}r_{Y|X}\left(y|x_{i}\right)\;\ln q_{Y|X}\left(y|x_{i}\right)\;dy+\sum_{i=1}^{n}\sum_{j\neq i}\int_{Y}q_{Y|X}\left(y|x_{j}\right)\;\ln q_{Y|X}\left(y|x_{j}\right)\;dy\right)\;dx^{n}\\ \; & =\frac{1}{n}[\;\sum_{i=1}^{n}\left\{\;\prod_{\ell \neq i}\int_{X}p_{X}\left(x_{\ell }\right)\;dx_{\ell }\cdot \int_{X\times Y}r_{Y|X}\left(y|x_{i}\right)\;\ln q_{Y|X}\left(y|x_{i}\right)\;p_{X}\left(x_{i}\right)\;dx_{i}\;dy\right\}\\ \; & \;+\sum_{i=1}^{n}\sum_{j\neq i}\left\{\;\prod_{\ell \neq j}\int_{X}p_{X}\left(x_{\ell }\right)\;dx_{\ell }\cdot \int_{X\times Y}p_{X}\left(x_{j}\right)\;q_{Y|X}\left(y|x_{j}\right)\;\ln q_{Y|X}\left(y|x_{j}\right)\;dx_{j}\;dy\right\}]\\ \; & =\frac{1}{n}\left[\;\sum_{i=1}^{n}\int_{X\times Y}r_{Y|X}\left(y|x\right)\;\ln q_{Y|X}\left(y|x\right)\;p_{X}\left(x\right)\;dx\;dy+\sum_{i=1}^{n}\sum_{j\neq i}\int_{X\times Y}p_{X}\left(x\right)\;q_{Y|X}\left(y|x\right)\;\ln q_{Y|X}\left(y|x\right)\;dx\;dy\right]\\ \; & =\int_{X\times Y}p_{X}\left(x\right)\;r_{Y|X}\left(y|x\right)\;\ln q_{Y|X}\left(y|x\right)\;dx\;dy+\left(n-1\right)\int_{X\times Y}p_{X}\left(x\right)\;q_{Y|X}\left(y|x\right)\;\ln q_{Y|X}\left(y|x\right)\;dx\;dy. \end{array}$$

### Appendix D.3. Proof of Equations (67)–(73)

(A64) $$\begin{array}{cc} p_{Y^{n}}\left(y^{n}\right) & =\int p_{Y^{n}|X^{n}}\left(y^{n}|x^{n}\right)\;p_{X^{n}}\left(x^{n}\right)\;dx^{n}\\ \; & =\frac{1}{n}\sum_{i=1}^{n}\left\{\prod_{j\neq i}\int q_{Y|X}\left(y_{j}|x_{j}\right)\;p_{X}\left(x_{j}\right)\;dx_{j}\cdot \int r_{Y|X}\left(y_{i}|x_{i}\right)P_{X}\left(x_{i}\right)\;dx_{i}\right\}\\ \; & =\frac{1}{n}\sum_{i=1}^{n}\left\{\prod_{j\neq i}v\left(y_{j}\right)\cdot w\left(y_{i}\right)\right\}\\ \; & =\prod_{j=1}^{n}v\left(y_{j}\right)\cdot \frac{1}{n}\sum_{i=1}^{n}\frac{w(y_{i})}{v(y_{i})},\;\forall \;y^{n}\in Y^{n}, \end{array}$$

where v(·) and w(·) are probability densities on Y, as defined in (68) and (69), respectively. This proves (67).

We next prove (70), which is used to calculate the entropy of $Y^{n}$ with the density $p_{Y^{n}}\left(\cdot \right)$ in (A64). In view of the integral representation of the logarithmic function in (1) and by interchanging the order of the integrations (due to Fubini’s theorem), we get that for a positive random variable *Z*

(A65) $$\begin{array}{cc} E\left\{Z\ln Z\right\} & =\int_{0}^{\infty }\frac{1}{u}\cdot E\left\{Z\;\left(e^{-u}-e^{-uZ}\right)\right\}\;du\\ \; & =\int_{0}^{\infty }\frac{E\left\{Z\right\}\;e^{-u}-E\left\{Ze^{-uZ}\right\}}{u}\;du\\ \; & =\int_{0}^{\infty }\frac{M_{Z}^{\prime }\left(0\right)\;e^{-u}-M_{Z}^{\prime }\left(-u\right)}{u}\;du, \end{array}$$

which proves (70). Finally, we prove (71). In view of (A64),

(A66) $$\begin{array}{cc} h(Y^{n}) & =-\int p_{Y^{n}}\left(y^{n}\right)\;\ln p_{Y^{n}}\left(y^{n}\right)\;dy^{n}\\ \; & =-\int \prod_{j=1}^{n}v\left(y_{j}\right)\cdot \frac{1}{n}\sum_{i=1}^{n}\frac{w(y_{i})}{v(y_{i})}\cdot [\;\ln\left(\;\prod_{j=1}^{n}v\left(y_{j}\right)\right)+\ln\left(\frac{1}{n}\sum_{i=1}^{n}\frac{w(y_{i})}{v(y_{i})}\right)]\;dy^{n}\\ \; & =-\int \prod_{j=1}^{n}v\left(y_{j}\right)\cdot \frac{1}{n}\sum_{i=1}^{n}\frac{w(y_{i})}{v(y_{i})}\cdot [\;\sum_{j=1}^{n}\ln v\left(y_{j}\right)+\ln\left(\frac{1}{n}\sum_{i=1}^{n}\frac{w(y_{i})}{v(y_{i})}\right)]\;dy^{n}\\ \; & =-\int \prod_{\ell =1}^{n}v\left(y_{\ell }\right)\cdot \frac{1}{n}\sum_{i=1}^{n}\sum_{j=1}^{n}\frac{w\left(y_{i}\right)\;\ln v\left(y_{j}\right)}{v(y_{i})}\;dy^{n}\\ \; & \;-\int \prod_{j=1}^{n}v\left(y_{j}\right)\cdot \frac{1}{n}\sum_{i=1}^{n}\frac{w(y_{i})}{v(y_{i})}\cdot \ln\left(\frac{1}{n}\sum_{i=1}^{n}\frac{w(y_{i})}{v(y_{i})}\right)\;dy^{n}. \end{array}$$

A calculation of the first integral on the right-hand side of (A66) gives

(A67) $$\begin{array}{cc} \int \prod_{\ell =1}^{n}v\left(y_{\ell }\right)\cdot \frac{1}{n}\sum_{i=1}^{n}\sum_{j=1}^{n}\frac{w\left(y_{i}\right)\;\ln v\left(y_{j}\right)}{v(y_{i})}\;dy^{n} & =\frac{1}{n}\sum_{i=1}^{n}\sum_{j=1}^{n}\int \prod_{\ell =1}^{n}v\left(y_{\ell }\right)\cdot \frac{w\left(y_{i}\right)\;\ln v\left(y_{j}\right)}{v(y_{i})}\;dy^{n}\\ \; & =\frac{1}{n}\sum_{i=1}^{n}\sum_{j=1}^{n}\int \prod_{\ell \neq i}v\left(y_{\ell }\right)\cdot w\left(y_{i}\right)\;\ln v\left(y_{j}\right)\;dy^{n}. \end{array}$$

For i=j, the inner integral on the right-hand side of (A67) satisfies

(A68) $$\begin{array}{cc} \int \prod_{\ell \neq i}v\left(y_{\ell }\right)\cdot w\left(y_{i}\right)\;\ln v\left(y_{j}\right)\;dy^{n} & =\prod_{\ell \neq i}\int v\left(y_{\ell }\right)\;dy_{\ell }\cdot \int w\left(y_{i}\right)\;\ln v\left(y_{i}\right)\;dy_{i}\\ \; & =\int w(y)\;\ln v(y)\;dy, \end{array}$$

and for i≠j,

(A69) $$\begin{array}{cc} \int \prod_{\ell \neq i}v\left(y_{\ell }\right)\cdot w\left(y_{i}\right)\;\ln v\left(y_{j}\right)\;dy^{n} & =\prod_{\ell \neq i,j}\int v\left(y_{\ell }\right)\;dy_{\ell }\cdot \int w\left(y_{i}\right)\;dy_{i}\cdot \int v\left(y_{j}\right)\;\ln v\left(y_{j}\right)\;dy_{j}\\ \; & =\int v(y)\;\ln v(y)\;dy. \end{array}$$

Therefore, combining (A67)–(A69) gives

(A70) $$\begin{array}{c} \int \prod_{\ell =1}^{n}v\left(y_{\ell }\right)\cdot \frac{1}{n}\sum_{i=1}^{n}\sum_{j=1}^{n}\frac{w\left(y_{i}\right)\;\ln v\left(y_{j}\right)}{v(y_{i})}\;dy^{n}=\int w\left(y\right)\;\ln v\left(y\right)\;dy+\left(n-1\right)\int v\left(y\right)\;\ln v\left(y\right)\;dy. \end{array}$$

Finally, we calculate the second integral on the right-hand side of (A66). Let $\mu_{n}$ be the probability density function defined as

(A71) $$\begin{array}{c} \mu_{n}\left(y^{n}\right):=\prod_{j=1}^{n}v\left(y_{j}\right),\;y^{n}\in Y^{n}, \end{array}$$

and let

(A72) $$\begin{array}{c} Z:=\frac{1}{n}\sum_{i=1}^{n}\frac{w(V_{i})}{v(V_{i})} \end{array}$$

where ${\left\{V_{i}\right\}}_{i=1}^{n}$ are i.i.d. Y-valued random variables with a probability density function *v*. Then, in view of (70), the second integral on the right-hand side of (A66) satisfies

(A73) $$\begin{array}{cc} \int \prod_{j=1}^{n}v\left(y_{j}\right)\cdot \frac{1}{n}\sum_{i=1}^{n}\frac{w(y_{i})}{v(y_{i})}\cdot \ln\left(\frac{1}{n}\sum_{i=1}^{n}\frac{w(y_{i})}{v(y_{i})}\right)\;dy^{n} & =E\left\{Z\ln Z\right\}\\ \; & =\int_{0}^{\infty }\frac{M_{Z}^{\prime }\left(0\right)\;e^{-u}-M_{Z}^{\prime }\left(-u\right)}{u}\;du. \end{array}$$

The MGF of *Z* is equal to

(A74) $$\begin{array}{cc} M_{Z}\left(u\right) & =\int_{Y^{n}}\prod_{i=1}^{n}v\left(r_{i}\right)\;\exp\left(\frac{u}{n}\sum_{i=1}^{n}\frac{w(r_{i})}{v(r_{i})}\right)\;d\underline{r}\\ \; & =\prod_{i=1}^{n}\int_{Y}v\left(r_{i}\right)\;\exp\left(\frac{u}{n}\;\frac{w(r_{i})}{v(r_{i})}\right)\;dr_{i}\\ \; & =K^{n}\left(\frac{u}{n}\right), \end{array}$$

where

(A75) $$\begin{array}{c} K\left(u\right):=\int_{Y}v\left(y\right)\;\exp\left(\frac{u\;w(y)}{v(y)}\right)\;dy,\;\forall \;u\in R, \end{array}$$

and consequently, (A75) yields

(A76) $$\begin{array}{cc} M_{Z}^{\prime }\left(u\right) & =K^{n-1}\left(\frac{u}{n}\right)\;K^{\prime }\left(\frac{u}{n}\right)\\ \; & ={\left(\int v\left(y\right)\;\exp\left(\frac{u\;w(y)}{v(y)}\right)\;dy\right)}^{n-1}\;\int w\left(y\right)\;\exp\left(\frac{u\;w(y)}{v(y)}\right)\;dy, \end{array}$$

and

(A77) $$\begin{array}{c} M_{Z}^{\prime }\left(0\right)=1. \end{array}$$

Therefore, combining (A73)–(A77) gives the following single-letter expression for the second multi-dimensional integral on the right-hand side of (A66):

(A78) $$\begin{array}{cc} \int \prod_{j=1}^{n}v\left(y_{j}\right)\cdot \frac{1}{n}\sum_{i=1}^{n}\frac{w(y_{i})}{v(y_{i})}\cdot \ln\left(\frac{1}{n}\sum_{i=1}^{n}\frac{w(y_{i})}{v(y_{i})}\right)\;dy^{n} & =\int_{0}^{\infty }\frac{1}{u}\;\left[e^{-u}-t^{n-1}\left(\frac{u}{n}\right)\;s\left(\frac{u}{n}\right)\right]\;du \end{array}$$

where the functions s(·) and t(·) are defined in (72) and (73), respectively. Combining (A66), (A70), and (A78) gives (71).

### Appendix D.4. Specialization to a BSC with Jamming

In the BSC example considered, we have X=Y={0,1}, and

(A79) $$\begin{array}{cc} \; & r_{Y|X}\left(y|x\right)=\varepsilon \;1\left\{x\neq y\right\}+\left(1-\varepsilon \right)\;1\left\{x=y\right\}, \end{array}$$

(A80) $$\begin{array}{cc} \; & q_{Y|X}\left(y|x\right)=\delta \;1\left\{x\neq y\right\}+\left(1-\delta \right)\;1\left\{x=y\right\}, \end{array}$$

where 1{relation} is the indicator function that is equal to one if the relation holds, and to zero otherwise. Recall that we assume $0<\delta <\varepsilon \leq \frac{1}{2}$. Let

(A81) $$\begin{array}{c} p_{X}\left(0\right)=p_{X}\left(1\right)=\frac{1}{2}, \end{array}$$

be the binary symmetric source (BSS). From (63) and (64), for u≥0,

$$\begin{array}{cc} f(u) & =\sum_{x,y}p_{X}\left(x\right)\;q_{Y|X}\left(y|x\right)\;\exp\left(-\frac{u\;r_{Y|X}\left(y|x\right)}{q_{Y|X}\left(y|x\right)}\right) \end{array}$$

(A82) $$\begin{array}{cc} \; & =\left(1-\delta \right)\;\exp\left(-\frac{(1-\varepsilon )u}{1-\delta }\right)+\delta \;\exp\left(-\frac{\varepsilon u}{\delta }\right), \end{array}$$

$$\begin{array}{cc} g(u) & =\sum_{x,y}p_{X}\left(x\right)\;r_{Y|X}\left(y|x\right)\;\exp\left(-\frac{u\;r_{Y|X}\left(y|x\right)}{q_{Y|X}\left(y|x\right)}\right) \end{array}$$

(A83) $$\begin{array}{cc} \; & =\left(1-\varepsilon \right)\;\exp\left(-\frac{(1-\varepsilon )u}{1-\delta }\right)+\varepsilon \;\exp\left(-\frac{\varepsilon u}{\delta }\right). \end{array}$$

Furthermore, we get from (A79), (A80), and (A81) that

(A84) $$\begin{array}{c} -\sum_{x,y}p_{X}\left(x\right)\;r_{Y|X}\left(y|x\right)\;\ln q_{Y|X}\left(y|x\right)=-\varepsilon \ln\delta -\left(1-\varepsilon \right)\ln\left(1-\delta \right)=d\left(\varepsilon ∥\delta \right)+H_{b}\left(\varepsilon \right), \end{array}$$

and

(A85) $$\begin{array}{cc} \; & -\sum_{x,y}p_{X}\left(x\right)\;q_{Y|X}\left(y|x\right)\;\ln q_{Y|X}\left(y|x\right)=H_{b}\left(\delta \right). \end{array}$$

Substituting (A82)–(A85) into (66) (where the integrals in (66) are replaced by sums) gives

(A86) $$\begin{array}{cc} H(Y^{n}|X^{n}) & =d\left(\varepsilon ∥\delta \right)+H_{b}\left(\varepsilon \right)+\left(n-1\right)\;H_{b}\left(\delta \right)+\int_{0}^{\infty }\left[f^{n-1}\left(\frac{u}{n}\right)\;g\left(\frac{u}{n}\right)-e^{-u}\right]\;\frac{du}{u}. \end{array}$$

Since the input is a BSS, due to the symmetry of the channel (55), the output is also a BSS. This implies that (in units of nats)

(A87) $$\begin{array}{c} H(Y^{n})=n\ln2. \end{array}$$

As a sanity check, we verify it by using (71). From (68) and (69), for y∈{0,1},

(A88) $$\begin{array}{cc} \; & v\left(y\right)=p_{X}\left(0\right)\;q_{Y|X}\left(y|0\right)+p_{X}\left(1\right)\;q_{Y|X}\left(y|1\right)=\frac{1}{2}, \end{array}$$

(A89) $$\begin{array}{cc} \; & w\left(y\right)=p_{X}\left(0\right)\;r_{Y|X}\left(y|0\right)+p_{X}\left(1\right)\;r_{Y|X}\left(y|1\right)=\frac{1}{2}, \end{array}$$

and from (72) and (73), it consequently follows that

(A90) $$\begin{array}{c} s\left(u\right)=w\left(0\right)\;\exp\left(-\frac{u\;w(0)}{v(0)}\right)+w\left(1\right)\;\exp\left(-\frac{u\;w(1)}{v(1)}\right)=e^{-u},\;\forall \;u\geq 0, \end{array}$$

and also

(A91) $$\begin{array}{c} t\left(u\right)=e^{-u},\;\forall \;u\geq 0. \end{array}$$

It can be verified that substituting (A88)–(A91) into (71) reproduces (A87). Finally, subtracting (A86) from (A87) gives (75).
